# Biogeographic patterns of modern benthic shallow-water molluscs and the roles of temperature and palaeogeographic legacy

**DOI:** 10.1038/s41598-025-06473-0

**Published:** 2025-07-01

**Authors:** Thomas A. Neubauer, Serge Gofas, Mathias Harzhauser

**Affiliations:** 1 SNSB – Bavarian State Collection for Palaeontology and Geology, Richard-Wagner-Straße 10, 80333 Munich, Germany; 2 Department of Earth and Environmental Sciences, Palaeontology & Geobiology, Ludwig-Maximilians-Universität München, Richard-Wagner-Str. 10, 80333 Munich, Germany; 3 Naturalis Biodiversity Center, Darwinweg 2, 2333 CR Leiden, The Netherlands; 4 Departamento de Biología Animal, Facultad de Ciencias, Universidad de Málaga, Campus de Teatinos, S/N, 29071 Málaga, Spain; 5 Institut Systématique Evolution Biodiversité (ISYEB), Muséum National d’Histoire Naturelle, 57 Rue Cuvier CP 51, 75005 Paris, France; 6 Geological-Paleontological Department, Natural History Museum Vienna, Burgring 7, 1010 Vienna, Austria; 7 Institut Für Erdwissenschaften, NAWI Graz Geocenter, Universität Graz, Heinrichstraße 26, 8010 Graz, Austria

**Keywords:** Biogeography, Historical legacy, Marine molluscs, Ocean circulation patterns, Provinciality, Sea-surface temperature, Biodiversity, Biogeography

## Abstract

Unveiling the processes that lead to biogeographic regionalisation is key to understanding the links between micro- and macroevolution, community processes and macroecology. However, many studies focus on present-day conditions while neglecting geological and palaeontological history. Here, we review the relevance of contemporary climatic conditions and ocean circulation patterns and their geological legacy on the distribution of marine benthic biota, using Mollusca as a model group. Based on global gridded occurrence data, we computed hierarchical cluster analyses and non-metric multidimensional scalings using Simpson’s distance index at three systematic ranks (species, genus, family). Generalised additive models were applied to assess the relationship between taxon distribution and global sea-surface temperature. In addition, we introduce a novel method to quantify the geographic coherence of clusters identified by cluster analysis to ascertain biogeographically meaningful interpretation. We show that contemporary climate and palaeogeographic changes, which have shaped ocean circulation patterns over geological time, have had a significant impact on the global distribution of benthic shallow-water marine molluscs. Our results indicate a high level of provincialism for species, slightly less so for genera, and a polar vs. circum-temperate–tropical structure for families. The biogeographic units defined by our cluster analyses match existing ocean currents for species, while the poorer regionalisation for genus- and family-level data is the result of geologically young seaways or land bridges. Our findings evidence the importance of considering historical processes for the biogeography of modern faunas.

## Introduction

Biogeographic regionalisation mirrors life’s response to past and current physical and biological forces^1^. It offers a framework to study the geographic relationships among biota in various spatial contexts and provides the basis for spatial studies on systematics, evolutionary biology or conservation^1,2^. Compared to our detailed knowledge of the biogeographic division of the world’s biota, less is known about the processes underlying these patterns, which roots in the complexity of natural processes and their interactions across geographic, taxonomic, and ecological scales^3^. Deciphering these mechanisms is, however, key to bridging the gap between microevolutionary and community ecology processes on the scale of individuals and populations (such as speciation, extinction, migration, selection, and biotic interactions) and macroevolution and macroecology.

The concept of biogeographic regionalisation reaches far back into the nineteenth century. For molluscs, Woodward^4^ already provided the first world map with “Molluscan Provinces”. Modern biogeographic concepts, in turn, are more holistic and often integrate a wide range of organisms. A highly pragmatic set of coastal realms, provinces, and ecoregions was published by Spalding et al*.*^2^, based on three principles: that they should have a strong biogeographic basis, offer practical utility, and minimize discrepancies with existing systems. Hill et al*.*^5^ defined bioregions as geographic regions that are relatively homogenous and distinct in terms of their biological contents and explored the theoretical aspects of different analytical methods. The most recent attempts to quantify the marine regions of the world across taxa were published by Costello et al*.*^6^ and Kocsis et al*.*^7^, who categorised biogeographic realms based on the statistical analysis of the distribution of various animal and plant clades.

Comparatively fewer studies elucidate the processes underlying global marine biogeographic patterns using a quantitative approach. Temperature is one of the key factors affecting marine life in various ways. On the individual–population level, it governs metabolic functions, fecundity, life cycle, survival rate, as well as species interactions^8–10^. On an evolutionary to macroecological scale, in turn, these processes influence rates of speciation and extinction, as well as migration^8,11^. A prime example is the impact of the current global warming, which has been repeatedly shown to have massive impact on marine biota across the planet^12–17^.

The relationship between temperature and patterns of biogeography and biodiversity have been commonly studied for species richness, often in the context of the latitudinal diversity gradient^9,17–22^, or body size^23,24^. Specifically concerning biogeographic provinciality, the impact of temperature has been discussed qualitatively by Spalding et al*.*^2^ and Costello et al*.*^6^. Using a statistical approach, Belanger et al*.*^18^ found that temperature is a strong predictor for existing biogeographic schemes. Similarly, Kocsis et al*.*^7^ provided a quantitative framework that evaluates the role of abiotic factors for biogeographic zonation, showing that temperature is the most important predictor of bioregion distributions. For bivalves, Tomašových et al*.*^21^ showed that species range sizes are inversely related to latitude and thus temperature.

The importance of geodynamics and the impact of changing palaeogeography on Neogene biogeography has been in the focus of many papers dealing with palaeontological data, both in the marine^25–30^ and continental realm^31–33^. This deep-time perspective, however, is only superficially considered in most contributions focusing on modern biogeography.

Here, we employ a combined approach to study global shallow-water marine provinciality and its drivers, assessing the potential influence of temperature and palaeogeography on biogeographic patterns, with molluscs as a model taxon. We gathered 3 million occurrence records of benthic shallow-water taxa from OBIS and GBIF across three systematic ranks (species, genera, families). Based on a 250 × 250 km equal-area grid, we use hierarchical cluster analysis to identify major biogeographic units and their hierarchical relationships across various levels of resolution. We apply multivariate ordination methods combined with generalised additive models to infer to which extent temperature drives global taxonomic compositions of benthic shallow-water molluscs. This approach is chosen to test for a relationship between biogeography and temperature independently of pre-defined units, in order to provide a picture unbiased by subjective interpretation. Biogeographic regions are matched with patterns of ocean circulation to explain boundaries. The links with temperature and ocean currents as well as faunistic distances among regions are interpreted in a palaeogeographic context to provide a long-term perspective on the biogeographic evolution of the world’s shallow-water marine biota.

## Results

**Fig. 1:**
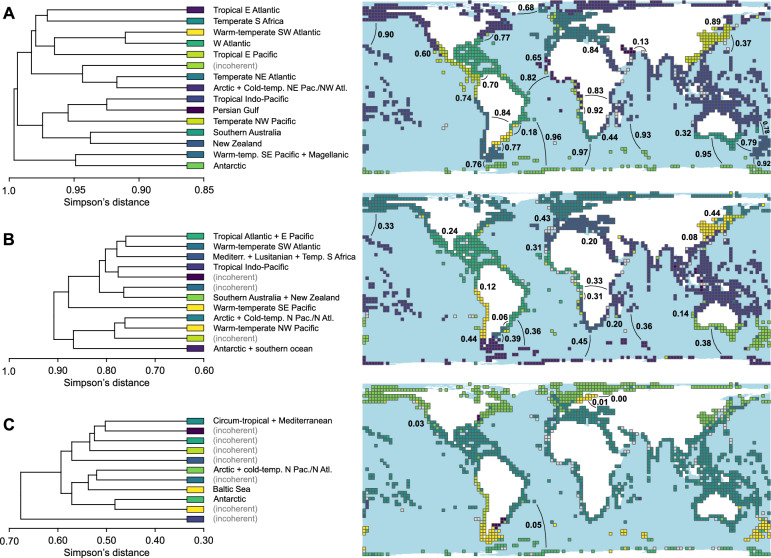
Simplified cluster dendrograms and distribution of clusters on the global map, for species (**A**), genera (**B**), and families (**C**) across a 250 × 250 km equal-area grid (Behrmann projection). Names of biogeographic units were chosen to match Spalding et al*.*^2^ as closely as possible. Clusters found to be incoherent according to the Cluster Coherence Index *I*_*CC*_ are marked as such (grey cells in the maps) and not discussed further. Simpson’s distances between adjacent clusters are indicated on the maps. Similarity cut-offs: 0.90 (**A**), 0.75 (**B**), and 0.48 (**C**); see Figure S8–16 in Supplementary Information 1 and Supplementary Information 2 for other trees and maps. Maps were created with R v. 4.3.2 and edited with CorelDRAW X8, https://www.coreldraw.com/.

Both the hierarchical cluster analyses and nMDS showed clear biogeographic structuring in the dataset, while highlighting different aspects. In the cluster analyses for all three systematic ranks, UPGMA outperformed WPGMA significantly according to the co-phenetic correlation coefficients (species: 0.814 over 0.733; genera: 0.709 over 0.594; families: 0.530 over 0.449). Accordingly, all subsequent analyses and dendrograms were based on UPGMA results. Our method to limit cut-off levels to an interval between at least ten larger clusters (≥ 5 cells) and ≥ 95% of the cells belonging to larger clusters constrained the relevant ranges of Simpson’s distances to 0.82–0.94 for species (four cut-off levels at a 0.04 distance interval), 0.67–0.76 for genera (three levels), and 0.44–0.50 for families (two levels). Here, we show for each systematic rank one representative map with simplified dendrogram, including Simpson’s distances among adjacent clusters (Fig. 1); see Supplementary Information 1 (Figure S8–16, Tables S1–18) for the additional figures and tables (see Supplementary Information 2 for full dendrograms).

The cluster dendrogram based on species data shows biogeographic structuring already at high Simpson’s distances. Already above a distance of 0.98, four main biogeographic units are identified, corresponding to 1) Antarctic, Southern Ocean, Warm-temperate Southeast Pacific, and Magellanic; 2) Tropical Indo-Pacific, Northwest Pacific, Southern Australian, and New Zealand; 3) Arctic, North Atlantic, and Northeast Pacific; 4) Tropical Eastern Pacific, Tropical Eastern Atlantic, Western Atlantic, and temperate South Africa (Fig. 1). With decreasing cut-off, the provincialisation becomes more refined, yet a few clusters remain stable down to lower cut-off threshold (0.82), i.e. Antarctic, West Atlantic, and Tropical Eastern Pacific (Figure S8–11). In contrast, Warm-temperate Southeast Pacific and Magellanic provinces split, as do Southern Australian and New Zealand, Arctic and Temperate Northeast Atlantic, Arctic and Northeast Pacific, Cold- and Warm-temperate Northwest Pacific (the latter including here the South China Sea), along with a more detailed regionalisation in the Tropical Indo-Pacific (e.g. Persian Gulf, Red Sea, and Tropical Southwestern Pacific; Figure S10–11). Few of the clusters were found to be geographically incoherent according to the Cluster Coherence Index* I*_*CC*_ (Fig. 1; Figure S8–11). Simpson’s (i.e. faunistic) distances among the clusters are high on average, ranging from 0.13 to 1 (only considering coherent and large clusters; Fig. 1; Figure S8–11, Table S10–13).

Note that the chosen cut-off levels preclude a more subtle subdivision, at least visually. Distinctions such as between the tropical western Atlantic and the temperate northwest Atlantic or between the tropical Panamanian region and the temperate northeast Pacific are traceable in the cluster analysis, but maps based on a corresponding cut-off level to make these clusters visible would mainly indicate clusters for well-sampled regions while excluding a large number of cells as outliers, particularly along the coasts of the southern Atlantic, Indian Ocean, and western Pacific.

The dendrogram based on genus data indicates two main clusters above a distance of 0.9, one including Arctic and Cold-temperate North Pacific and North Atlantic, Temperate Northwest Pacific, as well as Antarctic plus Magellanic (along with a few outliers in the Atlantic); the other one combining primarily tropical and warm-temperate faunas. Particularly noteworthy compared to the dendrogram based on species is the combined Tropical Eastern Pacific and Western Atlantic cluster (Fig. 1). At higher distances, this also includes the Tropical Eastern Atlantic. Mediterranean plus Lusitanian and temperate southern Africa form another cluster at higher distances (Fig. 1). Australian and New Zealand, Antarctic and Magellanic, as well as Antarctic and Southern Ocean split at lower distances, in addition to several sub-clusters in the Tropical Indo-Pacific (but less clearly than for species). The *I*_*CC*_ identified a number of smaller clusters geographically incoherent (Fig. 1; Figure S12–14). Across the constrained range of distances (0.67–0.76), Arctic, Warm-temperate Northwest Pacific, and Warm-temperate Southeast Pacific remain more or less stable. Simpson’s distances among the clusters are moderate, ranging from 0 to 0.83 (Fig. 1; Figure S12–14, Table S14–16; Supplementary Information 2).

The family data dendrogram shows a much coarser picture. Clusters diversify only below a Simpson’s distance of 0.6, and most of them are identified as geographically incoherent by *I*_*CC*_. The two primary clusters combine regions similarly as for genera, basically contrasting polar/cold-temperate regions and tropical/warm-temperate ones. An exception here is that New Zealand is combined with the Southern Ocean (including also Magellanic) and thus polar/cold-temperate cluster, while Southern Australia groups with tropical/warm-temperate faunas. At lower distances, Antarctic, Arctic, Baltic Sea, Southern Ocean, Warm-temperate Southwest Atlantic, and Warm-temperate Southeast Pacific constitute separate clusters (albeit the latter three are found geographically incoherent due to the presence of outliers), compared to a broad circum-tropical to warm-temperate cluster (Fig. 1). Simpson’s distances among the clusters are minor, ranging from 0 to 0.17 (Fig. 1; Figure S15–16, Table S17–18; Supplementary Information 2). The comparatively low values reflect the presence/absence of certain families rather than compositional differences.

**Fig. 2:**
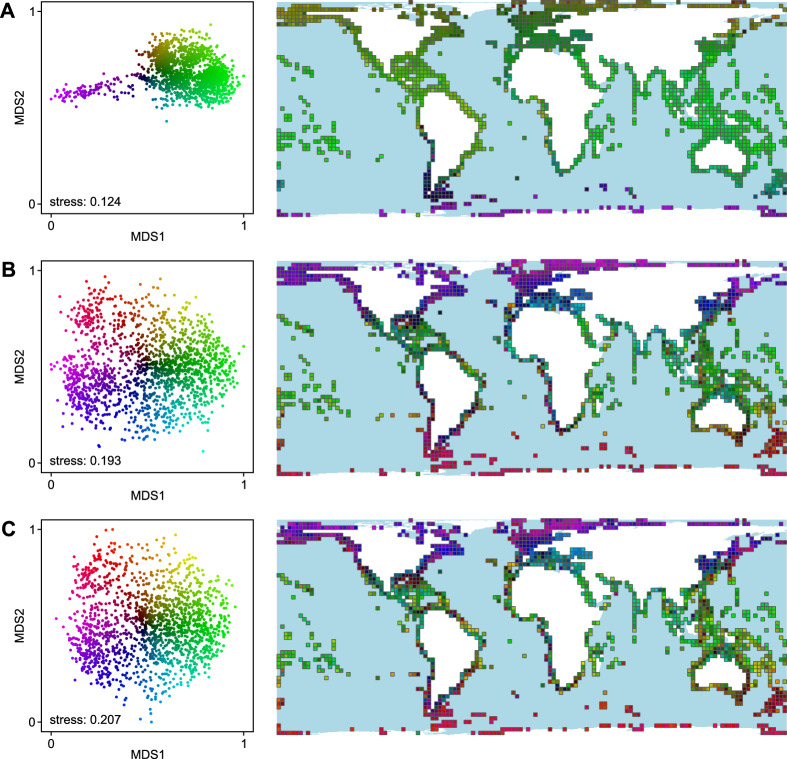
Results of the non-metric Multidimensional Scaling (nMDS) based on the Simpson’s dissimilarity matrices for species (**A**), genera (**B**), and families (**C**). Colours in the ordination plots refer to the position in the plot; grid cells in the maps are coloured accordingly to visualize similarities geographically. Plots are scaled and rotated to facilitate comparison (see Methods chapter for details). Cells with less than 10 occurrence records were omitted. Grid and projection as in Fig. 1. Maps were created with R v. 4.3.2 and edited with CorelDRAW X8, https://www.coreldraw.com/.

The nMDS of species, genus, and family level revealed various degrees of provinciality, yet without the clear-cut boundaries of clustering algorithms (Fig. 2). In all cases, faunas from polar regions were most distinct and showed a clear differentiation between Arctic and Antarctic faunas. The ordination based on species-level dissimilarities (stress: 0.124) also distinguished the faunas of the southeastern Pacific to southwestern Atlantic from circum-temperate–tropical faunas. The ordinations for genus- and family-level data show a much more differentiated and comparatively chaotic picture, probably owed to the difficulty of projecting a complex structure onto few dimensions; this is also mirrored in the comparatively higher stress values (0.193 and 0.207, respectively; Fig. 2; Figure S7).

**Fig. 3:**
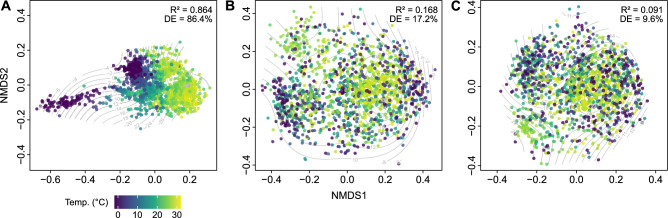
nMDS ordination plots with global sea-surface temperature fitted as smooth surface using a generalised additive model for species (**A**), genera (**B**), and families (**C**). Adjusted R^2^ and deviance explained (DE) indicate the strength of the relationship between temperature and ordination.

The results of the nMDS are mirrored in the generalised additive model with global sea-surface temperature. The model for species-level dissimilarities shows a strong match between biogeographic structure and temperature (R^2^ = 0.864, deviance explained: 86.4%; Fig. 3A). It is considerably lower for genera (R^2^ = 0.168, deviance explained: 17.2%; Fig. 3B) and virtually absent (R^2^ = 0.091, deviance explained: 9.6%; Fig. 3C) for families. The Mantel tests yielded similar results: only the test for species showed a significant correlation (r = 0.345, p = 0.001), while genera (r = −0.067, p = 1) and families (r = −0.154, p = 1) did not. The lower correlation for species compared to the high deviance signalled by the nMDS roots in the fact that the Mantel test assesses the total match between temperature and taxonomic compositions of the grid cells, while the nMDS is already constrained to the main taxonomic signal.

## Discussion

Our results offer new insights into the provincialism of the world’s shallow-water benthic molluscs across various scales. Using a comprehensive dataset and a new analytical approach without a priori defined units, we find evidence for the overarching importance of temperature for biogeographic structuring. Below, we discuss and show the persistent relevance of temperature, or more specifically, the linkage of molluscs to cold or warm water masses and their variation through geological time, on marine biogeography. Moreover, we find deviations of our new biogeographic scheme from previous ones and elaborate on the potential causes.

### The impact of temperature and ocean currents

All our analyses indicate latitudinal differences, with a strong distinction of polar versus circum-temperate–tropical faunas. This is especially evident from the nMDS (Fig. 2) and higher relationships among clusters of the hierarchical cluster analyses (Fig. 1). The latitudinal distinction is clearest for families and genera, but less so for species, which present a more refined regionalisation. These finer-scale differences in species composition are mirrored by the better fit in the analyses with global sea-surface temperature compared to genera and families (Fig. 3).

Our results for species are similar to what was found in a previous study on global shallow-marine benthic faunas^18^. These authors tested for the prediction power of temperature on fixed, existing biogeographic schemes, while our analyses are not based on any given boundaries or a priori assumptions.

The link with temperature reminds conspicuously of the latitudinal diversity gradient^8,9,19,20,34–37^. Various hypotheses have been brought forward to explain latitudinal differences in species richness and specifically how it relates to variation in temperature. Metabolic and speciation rates are higher, and generation cycles are shorter in warm climates, while extinction rates tend to be lower^8,11,35,36^. A higher demand in nutrients with increasing temperatures boosts biotic interactions such as competition and predation^9,10^.

Consequently, a smaller set of species is adapted to the cold waters of the world’s polar regions, which accounts not only for differences in species richness but also for the biogeographic signatures we observe here. Many of those species belong to genera and families that are also present in temperate and tropical areas. Conversely, many genera and families are consistently restricted to tropical regions. The comparatively weak relationship between taxonomic composition and temperature for genera and families shows that related species can be adapted to very different climatic regimes, causing many genera and families to occur in a broad range of temperatures. This is particularly the case for species-rich genera^38^.

**Fig. 4:**
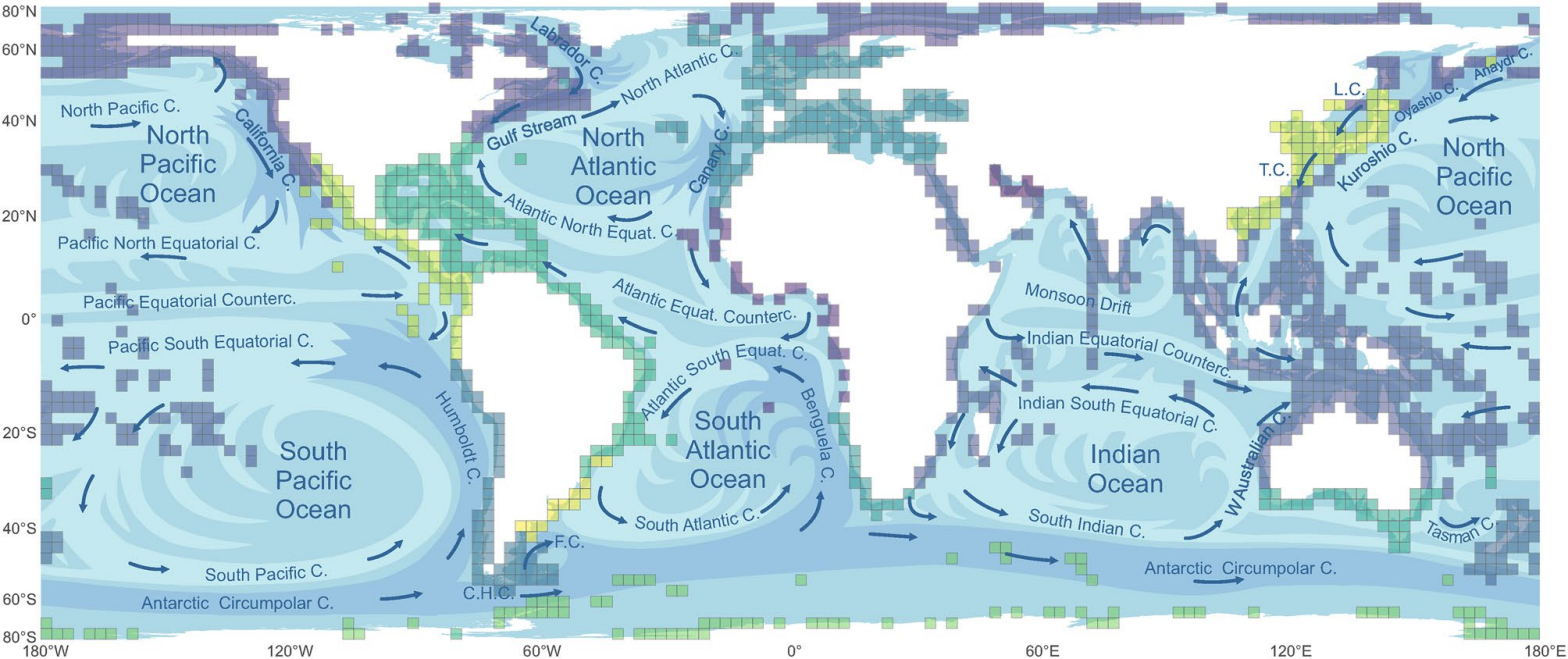
Biogeographic regions as defined by the hierarchical cluster analysis for species (compare Fig. 1A) and current ocean circulation patterns (modified from https://www.britannica.com/science/ocean-current). Light blue colours indicate warm currents, dark blue show cold currents. Note that directions for the Monsoon Drift here refer to Northern Hemisphere winter conditions; it alternates during summer. Incoherent clusters were omitted in this figure. C.H.C – Cape Horn Current; F.C. – Falkland Current; L.C. – Liman Current; T.S. – Tsushima Current. Map was created with R v. 4.3.2 and edited with CorelDRAW X8, https://www.coreldraw.com/.

Aside from the general latitudinal temperature pattern affecting biogeographic relationships on a larger scale, individual warm- and cold-water currents influence the distribution of benthic shallow-water molluscs and often contribute to sharpen the transitions. We find that the boundaries of many biogeographic regions identified by our cluster analyses coincide with the convergence of ocean currents (Fig. 4). Examples are numerous: the Warm-temperate Southeast Pacific coincides with the limits of the Humboldt (Peru) Current both in the north and the south. The Magellanic region links to the Cape Horn and Falkland currents. The Arctic-type faunas extend in the northwestern Atlantic down to mid latitudes due to the cold-water Labrador Current, and in the northeastern Pacific even further as a result of the California Current. Wedged in between the cold-water California Current in the north and the Humboldt Current in the south is the Tropical Eastern Pacific region supplied by warm Pacific equatorial currents. The North Atlantic Current brings warm water towards northern Europe, causing the distinction of European and Arctic faunas. The transition between Temperate Southern Africa and Tropical Eastern Atlantic regions coincides with the transition of the Benguela Current and the Atlantic South Equatorial Current. The Tropical Indo-Pacific region in the Indian Ocean is delimited in the south by the South Indian and West Australian currents. In the northwestern Pacific, the Liman, Oyashio, and Anadyr Currents bring cold water to the Japan Sea as well as northeastern Honshu, marking the transition between Cold- and Warm-temperate Northwest Pacific faunas (Fig. 4).

Partly, these patterns are traceable also for genus-level compositions, and in rarer cases also for family-level data. Particularly the Warm-temperate Southeast Pacific region (although it is not found coherent for family-level data because of outliers), the boundaries between Arctic and warm-temperate/tropical faunas, as well as between Antarctic and Southern Ocean/Magellanic faunas are visible across systematic ranks (Fig. 1, 4). This may in part be related to abrupt shifts in temperature regimes due to strong currents, such as the Humboldt Current pulling the equatorward limit of the temperate zone to the north. For genera, the delimitation of Northern Atlantic and European faunas differs from that for species, with northern European faunas showing a higher affinity towards Arctic than western European to Mediterranean faunas.

For the most part, these trends reflect large-scale climatic trends (polar vs. temperate/tropical), with the exception of the Warm-temperate Southeast Pacific region. Its persistence across all systematic ranks indicates that the Humboldt Current is an important driver of biogeographic distinction. This may root in its specific hydrological properties: because of intensive coastal upwelling, it is the most productive of all eastern boundary currents and has yielded more fish catch than any other ocean region in the world^39,40^. The Humboldt Current System is affected by a variety of seasonal to centennial fluctuations, such as the El Niño phenomenon^40^. As a result, the associated biogeographic unit, variably termed Warm Temperate Southeastern Pacific^2^, Humboldt Current Large Marine Ecosystem^41^ or Peruvian Province^42^, harbours a unique mollusc fauna.

### Ocean circulation, palaeogeography, and biogeography

An aim of this paper is to identify the influence of temperature and oceanographic history on the distribution of benthic molluscs. Yet, these factors cannot be considered separately, but they are in fact tightly interrelated. The reconfiguration of land bridges and seaways over the past million years has had severe effects on global ocean currents and, therefore, temperature^43–46^. Particularly the legacy of Cenozoic changes is still traceable nowadays. Many gateways, be it the opening of a seaway or the formation of a land bridge, are reflected in elevated biogeographic distances between clusters. In the following, we discuss the most important reconfigurations of gateways and ocean currents in the geological past, especially highlighting those that link to the boundaries of the here observed biogeographic units. The most important information is summarized in Table 1.

**Table 1:** Paleogeographic/oceanographic events referred to in the text, with information on geological ages and the biogeographic units involved. Note that the given biogeographic units primarily refer to the species-level results (compare Fig. 1A).

Paleogeographic/oceanographic event	Biogeographic unit(s) involved	Age	Notes	Source
Opening of Atlantic Ocean	Tropical Eastern Atlantic < > Western Atlantic	Early Jurassic to mid-Cretaceous	Age range indicates diachronous opening of North and South Atlantic	47, 48
Formation/intensification of Labrador Current	Western Atlantic < > Arctic + Cold-temperate Northeast Pacific/Northwest Atlantic	Latest Cretaceous to Miocene	Age range refers to potential onset versus significant intensification of current with influence on Gulf Stream	49
Split of Zealandia from Gondwana	New Zealand < > Tropical Indo-Pacific/Southern Australia/Antarctic	Late Cretaceous		50, 51
Opening of Drake and Tasmanian passages/formation of Antarctic Circumpolar Current	Antarctic < > Warm-temperate Southeast Pacific + Magellanic/Temperate South Africa/Southern Australia/New Zealand/Tropical Indo-Pacific	Latest Eocene to early Oligocene		52, 53
Formation of *Gomphotherium* land bridge	Tropical Indo-Pacific < > Temperate Northeast Atlantic	Early Miocene		54
Restriction of Indonesian Passage	Tropical Indo-Pacific	Early Late Miocene	Restricted connection but still open	59
Formation/intensification of Humboldt Current System	Warm-temperate Southeast Pacific + Magellanic < > Tropical Eastern Pacific/Warm-temperate Southwest Atlantic/Antarctic	Late Oligocene to latest Miocene	Age range concerns onset versus intensification, with increased input of cold water	42, 60
Closing of Central American Seaway/formation of Isthmus of Panama	Tropical Eastern Pacific < > Western Atlantic	Miocene to Late Pliocene	Age range mirrors shift from constrained deep ocean circulation to ultimate closure of seaway	61–64

A major distinction concerns Western and Eastern Atlantic tropical faunas, which show strong differences on the species level but comparatively little variation on the genus level (Fig. 1). In addition to geologically more recent events and reconfigurations of ocean circulations, this pattern in part reflects the old age underlying the opening of the Atlantic Ocean in the Mesozoic: The Northern Atlantic opened in the Jurassic^47^, the Southern Atlantic separated Africa and South America during the mid-Cretaceous^48^.

In the north, the Tropical Western Atlantic is delimited by the southward flowing cold-water Labrador Current. The onset of that circulation pattern (or proto-versions of it) is a matter of current debate; dates range from as early as the latest Cretaceous to the Miocene^49^. The old age is mirrored in high species-level differences between the Tropical Western Atlantic and Arctic faunas, being almost as high as between Western and Eastern Atlantic tropical faunas.

Similarly, New Zealand faunas were found clearly distinct in most analyses. The continent of Zealandia diverged from Gondwana back to the Late Cretaceous^50^, leading to long-term isolated faunal development of shallow marine biota^51^, which explains at least in part the observed faunistic separation.

The full opening of the Drake and Tasmanian passages to deep oceanic circulation during the latest Eocene to early Oligocene led to the formation of the Antarctic Circumpolar Current^52,53^. In addition to polar temperatures, this old oceanographic system explains the high faunal distances of the Antarctic to any other faunal region. On average, distances are higher than between the Arctic and adjacent regions.

The formation of the *Gomphotherium* land bridge between Africa and Europe closed the Tethys Seaway in the Early Miocene^25^. Faunistic connectivity for benthic molluscs ceased even several million years before, causing an isolated development of Indian Ocean and Mediterranean faunas since ca. 21 Myr ago^54^. Combined with the different thermal regimes (tropical versus warm-temperate), this contributes to the high faunistic distance between the respective clusters in our analyses. The current anthropogenically induced biotic immigration from the Indo-Pacific into the Mediterranean since the opening of the Suez canal in 1869, as well as continuing global warming^55–58^, tend to attenuate this differentiation.

The Indonesian Passage between the Indian and Pacific oceans has been restricted from approximately 11 Myr ago (early Late Miocene) onward but has remained open until today^59^. This rather young geological tendency may explain why the Tropical Indo-Pacific is still a rather homogenous cluster for species and genera, despite major diversification of this tropical diversity hotspot already since the Miocene^27,30^. Faunistic differences within the Tropical Indo-Pacific relate to restricted seas (e.g. Red Sea, Persian Gulf) or subregions, such as the northern Indian Ocean coastlines, rather than a distinction between Pacific and Indian Ocean faunas (Fig. 1).

The Humboldt Current System, leading to the particular biogeographic signature discussed above, is a rather young geological feature in its present form. While the characteristic upwelling signatures have prevailed at least since the Late Oligocene, an increased contribution of cold Antarctic waters starting ca. 6 Myr ago coincided with major turnovers in the Miocene and Pliocene^42,60^. This is also reflected in our analyses: the region’s fauna is closely related to Antarctic-type species assemblages.

The faunistic distance between the Tropical Western Atlantic and Eastern Pacific has its origins in the younger geological history of the region: the closing of the Central American Seaway and formation of the Isthmus of Panama accordingly. The gateway was ultimately closed by ca. 2.8 Ma^61^, but deep ocean circulation had been constrained already since the Miocene^62–64^. This strongly increased both the intensity and seasonality of the North Atlantic Current and Gulf Stream considerably^62^. This geologically young development had an impact on species distributions but apparently not (yet) on genus-level similarities, which show no distinction among Pacific and Atlantic faunas (Fig. 1).

Apart from the geological time these ocean circulation patterns have existed, variation of their intensity and seasonality throughout Earth history, and particularly during the Quaternary icehouse climate (e.g. related to Milankovitch-scale climatic oscillations), have had additional impact on the composition and distribution of benthic marine life – including but not limited to molluscs. Going into detail is beyond the scope of this globally-focused study, but examples exist^65–68^. Future studies may test for a correlation between faunistic distances and the evolutionary age of modern faunas, particularly under the consideration of fossil assemblages.

### Previous biogeographic studies – where do molluscs diverge?

The biogeographic zonation constructed by the cluster analyses largely mirrors existing studies based on a larger set of marine organisms^2,6,7^. Our results are particularly similar to those of Kocsis et al*.*^7^, based on a network analysis of a dataset containing bryozoans, brachiopods, bivalves, gastropods, zooxanthellate stony corals, echinoderms, and decapods. For example, their regions termed Arctic, European, Tropical East Atlantic, South African, Tropical East Pacific, Tropical Indo-Pacific, Temperate Australian, and New Zealandian match the regions in our species-level cluster analyses (Fig. 1A) almost perfectly. Differences are noted for the Warm-temperate Southeast Pacific, which is delimited in our analyses as cluster separate from the Antarctic but not distinguished by Kocsis et al*.*^7^, and the Cold-temperate Northwest Pacific, which we find to be part of the cluster also including the Yellow Sea.

Costello et al*.*^6^ used a different approach using pre-defined sea areas as well as a larger dataset, including additionally selected groups of algae, annelids, nematodes, sponges, and fish. While a few selected regions, e.g. the Tropical East Pacific, Southern Africa, Southern Australia, New Zealand, and Antarctica, largely match ours, the majority does not. The empirical zonation presented by Spalding et al*.*^2^ matches largely our regions, but the borders differ when observed in detail. Also when considering studies on specific mollusc clades or biogeographic regions^18,69^, the results slightly deviate from ours, but yet again this may relate to different data sources or analytical approaches.

Especially since the results of Kocsis et al*.*^7^ largely match ours, we expect that the considerable discrepancies compared to other studies either root in the different methodology or the type of faunas included or both, rather than assuming a major difference of the biogeographic signals of molluscs compared to other groups. Particularly, the inclusion of nektonic and planktonic taxa by Spalding et al*.*^2^ and Costello et al*.*^6^ certainly affects the broader picture, since taxa with these types of lifestyle differ markedly in their dispersal potential.

### Limitations

Studies like this one relying on large databases come with certain limitations. While the amount of data stored in OBIS and GBIF is impressive, the data represent unbalanced input combining many different types of source and concepts. We have identified a number of points that potentially causes variation in the dataset, but we do not expect a major bias of our results and conclusions.

Geographic coverage: varying research focus and intensity over the past centuries and different efforts to make data available via online portals among researchers have led to considerable geographic variation in the amount and spread of occurrence data available through OBIS and GBIF^70,71^. In our particular case, the shallow-water regions off North America, northern Europe, Australia, and New Zealand have provided large sets of occurrences to these databases and are overrepresented in the dataset (Figure S1). Additionally well covered are the shores of countries like Japan, China, South Korea, South Africa, as well as certain hotspots such as the Philippines, New Caledonia, Hawai’i, and many other tropical island archipelagos. Comparatively poor is the record for parts of the southern Atlantic, especially along western Africa, the western and northern Indian Ocean, as well as coast segments in the Arctic and Antarctic (Figure S1). While we do not expect polar faunas to yield significantly higher diversity or different taxonomic compositions with more intensive sampling, the underrepresentation of tropical to subtropical regions in the Atlantic and Indian oceans most likely has an effect on the observed biogeographic patterns (compare Ondo et al*.*^72^ for geographic biases in plants and Borgelt et al*.*^73^ for a conservation perspective). Probably, this bias contributed to the limited capability of the cluster analyses to find clear biogeographic boundaries in the Southeastern Atlantic^69^ or allowing for a more refined hierarchy of clusters in the Tropical Indo-Pacific. Future research and data mining effort should be directed toward filling these gaps.

Taxonomic resolution and accuracy: a large portion of the occurrence records only provide genus or family names (9.6% and 2.6%, respectively). In other cases, the reliability of identifications is questionable. The latter is especially true for unrevised museum collections bearing partly outdated identifications or widely known misidentifications that have not been accounted for. All these aspects concur with a seriously declining taxonomic expertise over the past decades^74–76^. We fear that this detrimental trend will continue to threaten data quality also in coming years. Without the input of experts, high-resolution studies on the species level will become increasingly difficult and unreliable.

Taxonomic currentness: many clades have not been revised in the past decades or generally remain poorly known because of small body size or identification problems (e.g. species with few diagnostic characters). This problem affects entire groups such as mega-diverse families of microgastropods (e.g. Triphoridae, Eulimidae, Pyramidellidae, or the conoidean families Mangeliidae and Raphitomidae), or nondescript bivalves such as protobranchs or Thyasiridae^77–79^.

Taxonomic practice: different schools around the world have used different taxonomic and systematic approaches and conventions and continue to do so, causing larger datasets to host a convolute of inconsistent and partly contrasting concepts^80^.

Methodology: most species are still identified purely based on external characters rather than anatomical, genetic or genomic data or a combination thereof, leading to species being defined using different species concepts. This variation has potentially huge impact on studies of biodiversity, biogeography, and conservation biology^80,81^.

Unknown diversity: several studies over the past years have stressed the importance of cryptic diversity and the potentially huge amount of undetected cryptic species^82–84^.

Technical issues: inconsistencies and variation in data quality in huge databases such as OBIS and GBIF have been shown to be potentially problematic for biodiversity analyses^85–87^. These link particularly to varying precision or erroneous records of geographic coordinates, partly as a function of georeferencing or projection issues, records of marine taxa on land or vice versa, and records of extinct taxa or still living taxa in the fossil record not marked as such. Although we have followed existing pipelines to account for such problems^85,86^ (see Methods chapter), a number of erroneous records may have been overlooked.

Environmental factors: here, we focus on temperature as a prime factor explaining differences in taxonomic composition. In the future, also other factors, such as seasonal temperature variation, nutrients, spatial complexity, and the nature of predominant substrate, may be taken into consideration to further specify potential drivers of biogeographic structure.

Lacking quantitative framework to explain the relationship between compositional differences among larger regions and the age of a connecting seaway or disrupting land bridge: Unfortunately, the paucity of such gateways limits the possibility of a reliable statistical inference. In addition, the ages available for many of them are poorly constrained or involve long temporal ranges of uncertainty. A similar problem concerns the ages of ocean currents, some of which have existed for millions of years but may have significantly shifted in their intensity through geological time (see e.g. discussion on the Humboldt Current). As such, not their onset but their intensification may have sparked biogeographic provincialisation. More data and better age constraints are needed to allow for a reliable quantitative assessment.

Despite all these technical and conceptual biases, we are confident that our interpretations hold true on the global scale. Nonetheless, we encourage further research to fill the abovementioned gaps to limit potential biases for future studies and allow a more refined picture of biogeographic relationships.

### Conclusion and outlook

Our findings show the importance of selected factors for biogeographic structure. Temperature and palaeogeographic legacy, the latter adding east–west barriers to the persistent north–south temperature thresholds, both have had a significant impact on benthic shallow-water marine life. More precisely, temperature explains a significant proportion of benthic mollusc biogeographic relationships. This association is mirrored by the match between the biogeographic units defined by our cluster analyses and present-day ocean circulation patterns. The similarities among biogeographic units at least partly relate to palaeogeographic history and associated changes in ocean currents. While we lack sufficient data for a quantitative approach, older palaeogeographic events tend to correspond to higher distances. However, in part this is also the result of different temperate regimes. Future studies may focus on disentangling the temperature versus legacy effect and their individual contributions to the observed biogeographic patterns, as well as include additional factors.

Our results have various implications. First, our analyses on three systematic levels indicate major differences in the biogeographic relationships and the influence of temperature on them. Considering the high-resolution picture presented by the species-level data compared to the genus or family level, we emphasize the need of utilizing species data, both for extant and fossil studies, for meaningful and detailed results. This also entails the pressing need for more taxonomic experts being able to identify taxa to the species level – 12.2% of the taxon records in the final dataset are identified on genus and family level only.

Second, the rapid climate change nowadays entails massive alterations of sea-surface temperature that strongly affect ocean currents. A prime example is the predicted break-down of the Atlantic Meridional Overturning Circulation within the century^88,89^. Direct or indirect anthropogenic activity also impacts the observed biogeographic structure in other ways, e.g. through habitat destruction, pollution, overfishing, or the introduction of invasive species^55,90^. Not only do these stressors affect the boundaries of biogeographic units, they ultimately threaten the existence of unique ecosystems, faunas, and species, many of which have a long and rich legacy. The strong relationship with temperature we show stresses the urgent need for countermeasures against climate change to avoid shifts in biogeographic structure of marine benthic life on earth. The picture we draw here based on molluscs is readily transferrable to other benthic groups.

## Methods

Taxon occurrence data were downloaded from the Global Biodiversity Information Facility (GBIF, https://www.gbif.org/) on 5 August 2024 and the Ocean Biodiversity Information System (OBIS, https://obis.org/) on 2 August 2024^91,92^. Data were filtered to include only non-fossil specimens, records containing coordinates, and taxonomic ranks equal or below the family. Given the problems associated with many records stored in such large databases^86^, a number of additional filters were applied: records with a coordinate uncertainty of > 100 km kilometres, with coordinates falling outside the earth’s range, those located precisely at the meridian or equator (using R package CoordinateCleaner v. 3.0.1^93^), as well as those dating from earlier than 1900 were excluded. Records on land were excluded, whereas we used a buffer of 0.1 degrees on the coastline to account for shoreline records with imprecise coordinates, using R packages geodata v. 0.6–2^94^, sf v. 1.0–16^95^, and terra v. 1.7–71^96^. The OBIS and GBIF datasets were combined, duplicates removed, and subsequently matched against the World Register of Marine Species^97^ using the R package worrms v. 0.4.3^98^ to account for latest updates in systematic placement, rank and taxonomic status. Nomina nuda, nomina dubia, taxa inquirenda, or otherwise uncertain names, as well as non-marine taxa were excluded. Marine groups with primarily planktonic, nektonic, and neustic adult life stage were excluded as well; this concerns Cephalopoda and among gastropods, the order Pteropoda, the nudibranch families Glaucidae and Phylliroidae, the caenogastropod families Atlantidae, Carinariidae, and Pterotracheidae, and the genera *Fiona* (nudibranch), *Janthina*, and *Recluzia* (Epitoniidae). The final combined dataset contained 3,189,399 taxon records for 30,847 species (2,799,177 records), 4,488 genera (306,307 records), and 524 families (83,915 records) (Supplementary Information, Figure S1).

A 250 × 250 km equal-area grid was generated to structure the dataset into equal bins and account for uneven sampling (see Supplementary Information for discussion on the choice of the grid size). The grid was intersected with the GEBCO_2023 global elevation model (resolution of 15 arc-seconds) to delimit the dataset to shallow-water records (≤ 200 m; Figure S2)^7^. For each systematic rank (species, genus, family), a pipeline of analyses and tests were run, following the basic outline of Kreft & Jetz^1^.

To evaluate differences between taxon communities in grid cells, we calculated the Simpson’s dissimilarity index among all cells using the R package betapart v. 1.6^99,100^. This measure is equivalent to the turnover component of the Sørensen-Dice coefficient and specifically focuses on compositional differences. The nestedness component, related to differences in taxonomic richness, is disregarded here.

Dissimilarities matrices were subjected to a non-metric multidimensional scaling (nMDS)^1,101^, calculated with the R package vegan v. 2.6–4^102^, whereas cells with less than 10 occurrences were omitted. Furthermore, we excluded cells with a mean dissimilarity index of > 0.99 or < 0.01, indicating extreme and potentially incorrect outliers. Each nMDS was run for 100 iterations to find a stable solution. We chose different numbers of dimensions for the three datasets of species (k = 2), genera (k = 3), and families (k = 4), since a lower number yielded results with high uncertainty (stress values >> 0.2) for genera and families.

nMDS scores were scaled between 0 and 1, rotated and colour-coded to allow for a better comparison of the results of the three datasets. A colour was assigned to each point (grid cell) in the ordination plot based on its scores, using the R package colors3d v. 1.0.1^103^, with a square-rooted radial colour gradient. The same colours were assigned to grid cells to illustrate compositional similarities on the global map.

To assess the relationship between biogeographic patterns and temperature, we used global sea-surface temperature (SST) rasters made available by Cao et al*.*^104^. This dataset provides monthly SST temperatures for a spatial resolution of 0.041° from July 2002 to December 2019. With the R package terra we combined all rasters files into a single, averaged raster (Figure S3) and extracted mean SST value per grid cell containing data (Figure S4). We used Mantel tests to compare taxonomic dissimilarity and temperature differences (Euclidean distances) among grid cells for each of the three systematic ranks. In addition, we fitted a generalised additive model as smooth surface of penalised splines to each of the nMDS ordinations of species, genus, and family dissimilarities. Since the nMDS reduce the dimensionality to the primary axes explaining most of the taxonomic variation in the dataset, these additional analyses help constraining the relationship between taxonomic composition and temperature to the main signal. As measures of the match between temperature and ordination we noted both the adjusted R^2^ and deviance explained.

The dissimilarity matrix was also input to a hierarchical cluster analysis computed with the R package vegan. We tested two agglomeration methods that were previously found to give the most accurate results^1^, unweighted pair-group method using arithmetic averages (UPGMA) and weighted pair-group method using arithmetic averages (WPGMA). We assessed the validity of the results using the co-phenetic correlation coefficient^1^.

Dendrograms were created with R package dendextend v. 1.17.1^105^ with a number of different cut-off levels. To avoid a subjective choice while covering a wide range of clustering resolution, we constrained the upper cut-off threshold to yield ten larger clusters (i.e. with at least 5 cells) and the lower threshold so that ≥ 95% of the cells belonged to larger clusters. Between these thresholds, we selected as many cut-off levels as would fit at a 0.04 similarity interval. Clusters were randomly colour-coded to facilitate distinction on the global maps; clusters with less than 5 cells were coloured grey and not considered any further. Cluster dendrograms were simplified based on the cut-off levels for illustration and interpretation purposes. For each cut-off level and systematic rank, we noted the core stats (number of species, degree of endemism) for each cluster and calculated Simpson’s dissimilarity matrices among all clusters (Supplementary Information 1).

### Cluster coherence index

Quantifying the geographic coherence of cells in a cluster is vital to interpret its relevance for biogeographic interpretation; if cells are widely dispersed, a biogeographic signal is unlikely, while touching cells may indicate faunal connectivity. To quantify the geographic coherence of clusters and find an objective way to assess their relevance for biogeographic interpretation, we developed a new metric, the Cluster Coherence Index *I*_*CC*_. The index is composed of two aspects, a metric based on geographic distance and one based on neighbourhood of a focal cell. This combination is necessary to account for the uneven nature of the biogeographic clusters assessed here. An index entirely based on distance would penalize elongate shapes of clusters (e.g. along coastlines), while a metric only quantifying the presence of neighbouring cells would be unable to measure the distance of gaps and thus detect outliers. The combined Cluster Coherence Index *I*_*CC*_ is quantified as

$${I}_{CC}=\sqrt{\frac{\overline{d }+\overline{d }(1-\widehat{N})}{e \sqrt{c}}}$$

where *c* is the number of cells of a given cluster, $\overline{d }$ is the mean geographic distance among all the cluster’s cell centroids, *e* is the cell edge length, and $\widehat{N}$ is the proportion of cells that have a cell belonging to the same cluster in a 3 × 3 neighbourhood. Geographic distances were computed with the function distm() of the R package geosphere v. 1.5–18^106^ using Haversine distances. For that, cell centroids were projected into a WGS84 projection.

Small *I*_*CC*_ values denote a high cluster coherence. For clusters without disjunct cells ($\widehat{N}$ = 1), i.e. all cells touching at least diagonally, *I*_*CC*_ scales with distance, for those consisting only of disjunct cells ($\widehat{N}$ = 0), *I*_*CC*_ scales with twice the distance. Clusters with a small mean geographic distance and without any disjunct cells would yield an *I*_*CC*_ < 1 (Figure S6). An *I*_*CC*_ > 1 indicates the presence of outliers, and with *I*_*CC*_ > 2 we consider a cluster as incoherent.

Note that the index is unitless and thus works for any input distance unit. Moreover, it is scaled for cell size to allow application to various geographic scales. We have run tests with several hypothetical cluster arrangements and cell sizes (Supplementary material, Figs S5–6). *I*_*CC*_ values remain relatively constant across most of the range of tested cell sizes, only above an edge length of 10^5^ km values depart distinctly from the average (Figure S6). Since such large cell size are, however, unusual and hardly useful in a biogeographic context, we consider the index robust against variation in cell size within reasonable limits. Finally, note that the index is constructed for grids with square cells, it may need to be adjusted if used for other formats.

All analyses were carried out in R v. 4.3.2^107^.

## Supplementary Information

Supplementary Information 1.

Supplementary Information 2.

## Data Availability

All results are presented in the main paper and the Supplementary Information. The underlying dataset is available at https://doi.org/10.57756/gmssv4.
