# Supplementary material for: Biogeographic patterns of modern benthic shallow-water molluscs and the roles of temperature and palaeogeographic legacy
Source: Sci Rep. 2025 Jul 1;15:20304. doi: 10.1038/s41598-025-06473-0 (PMC12217645; doi:10.1038/s41598-025-06473-0)
Supplement: Supplementary file 1 — Supplementary Information 1. [file 41598_2025_6473_MOESM1_ESM.pdf]

# Biogeographic patterns of modern benthic shallow-water molluscs and the roles of temperature and palaeogeographic legacy

Supplementary Information 1

Thomas A. Neubauer[1,2,3]*, Serge Gofas[4,5], Mathias Harzhauser[6,7]

[1]SNSB – Bavarian State Collection for Palaeontology and Geology, Richard-Wagner-Straße 10, 80333 Munich, Germany

[2]Department of Earth and Environmental Sciences, Palaeontology & Geobiology, Ludwig-Maximilians-Universität München, Richard-Wagner-Str. 10, 80333 Munich, Germany

[3]Naturalis Biodiversity Center, Darwinweg 2, 2333 CR Leiden, The Netherlands

[4]Departamento de Biología Animal, Facultad de Ciencias, Universidad de Málaga, campus de Teatinos, s/n, E29071, Málaga, Spain

[5]Institut Systématique Evolution Biodiversité (ISYEB), Muséum National d'Histoire Naturelle, 57 rue Cuvier CP 51, 75005 Paris, France

[6]Geological-Paleontological Department, Natural History Museum Vienna, Burgring 7, 1010 Vienna, Austria

[7]Institut für Erdwissenschaften, NAWI Graz Geocenter, Universität Graz, Heinrichstraße 26, 8010 Graz, Austria

*Corresponding author: neubauer@snsb.de

## Methods

### Choice of grid size

Various different grid sizes are being used in biogeography studies. Costello *et al.* [1] used an approach with 5° c-squares, representing an edge length of ca. 550 km. Kocsis *et al.* [2] applied a hexagonal grid with a cell area of approximately 65,000 km², roughly equivalent to our 250 km square grid. To find an optimal grid size we performed preliminary tests. We found that a coarse grid, such as the one applied by Costello *et al.* [1], limits the level of detail that can be inferred; smaller biogeographic units may simply be overlooked. Also, it severely impacts the resolution of the analyses on the relationship between biogeographic structure and temperature and ocean currents, and accordingly, the possible interpretations. Moreover, in a coarser grid, many a cell would include more than one geographic realm (e.g., at the Strait of Gibraltar or the Isthmus of Panama).

On the other hand, a too high resolution causes many grid cells to lack data, or causes broadly sympatric species to occur in adjacent but distinct cells. For example, using a 100 x 100 km grid on our species dataset would result in 40.1% of the cells having no data. To some extent, this may be a result of excluding records with too high coordinate uncertainties (> 100 km), on the other hand some areas are indeed poorly sampled or are at least not well covered by OBIS and GBIF; some cells may only have records on genus or family level.

Our choice of 250 x 250 km (62,500 km²) is an intermediate solution, with fewer than 10% of the cells missing data (9.8%) while maintaining a sufficient resolution to reconstruct biogeographic units and make inferences about the relationship with temperature and ocean circulation patterns.

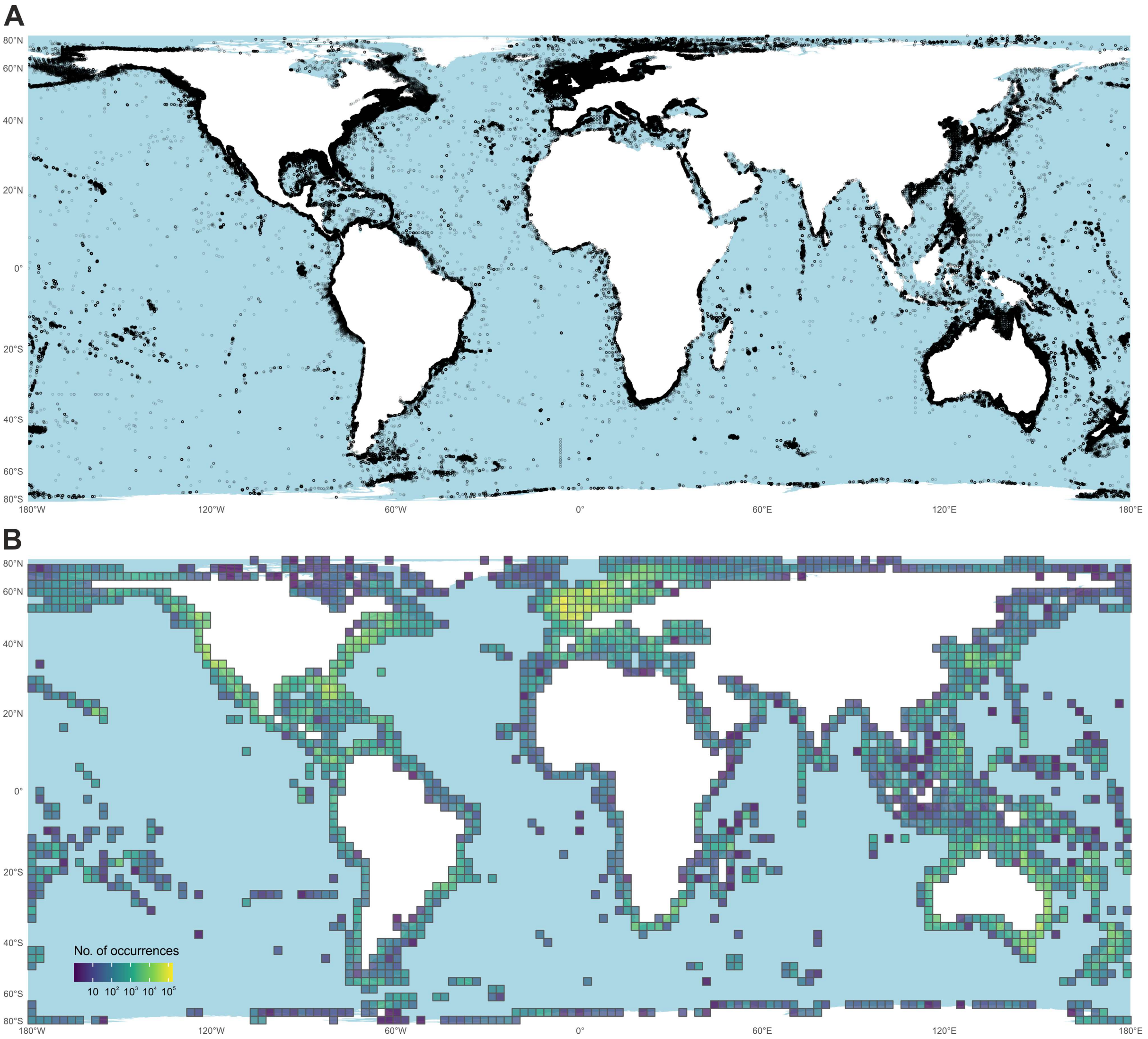


**Figure S1.** Combined occurrence dataset of 3,189,399 records of marine benthic shallow-water Mollusca stored in GBIF [3] and OBIS [4], shown as individual points (A) as well as a heat map giving the log-transformed number of occurrences per grid cell (B). Note the widely discrepant sampling intensities.

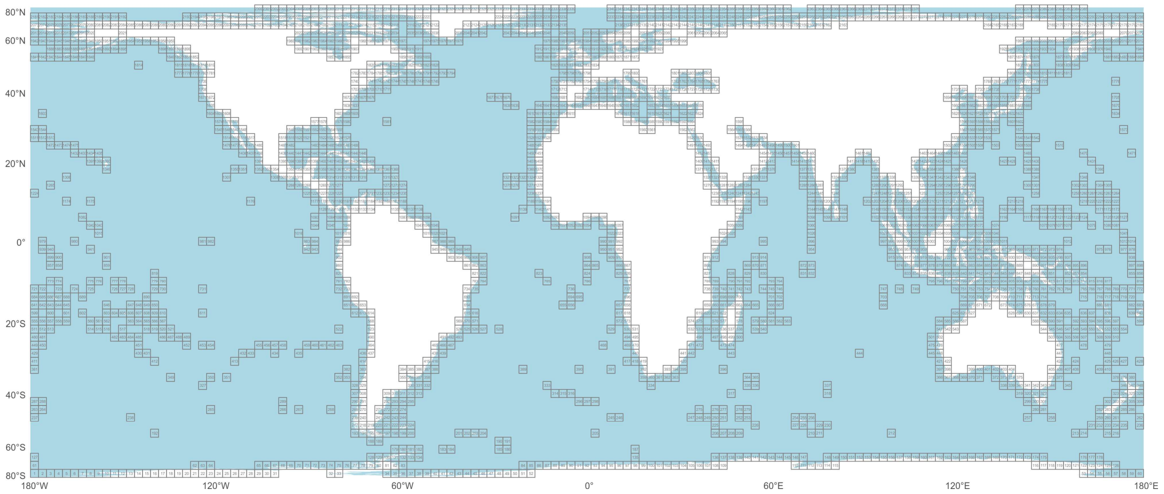


**Figure S2.** 250 x 250 km grid with cell IDs. Projection: Behrmann (equal-area).

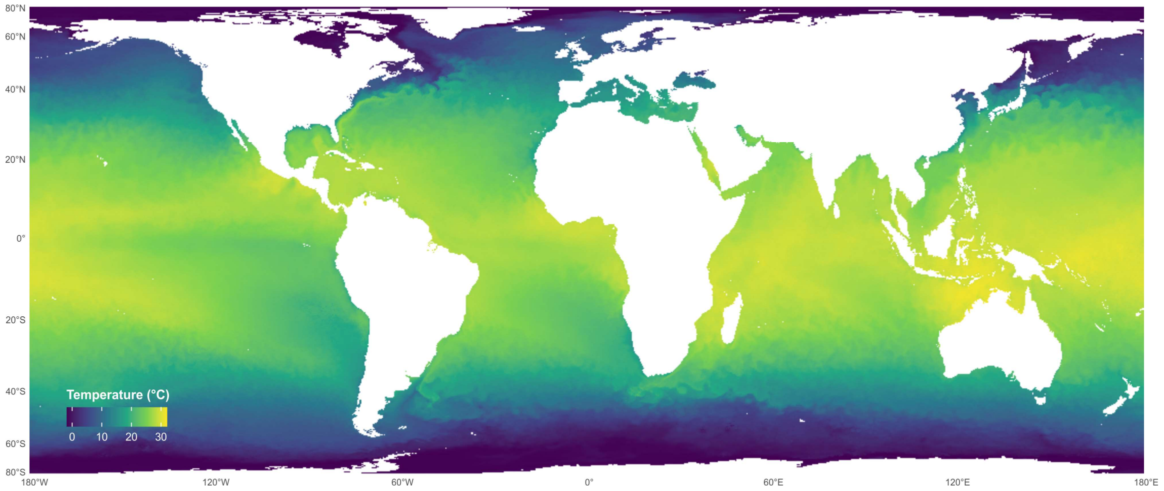


**Figure S3.** Global sea-surface temperature raster. The map represents the mean of monthly rasters from July 2002 to December 2019 at a spatial resolution of 0.041° [5].

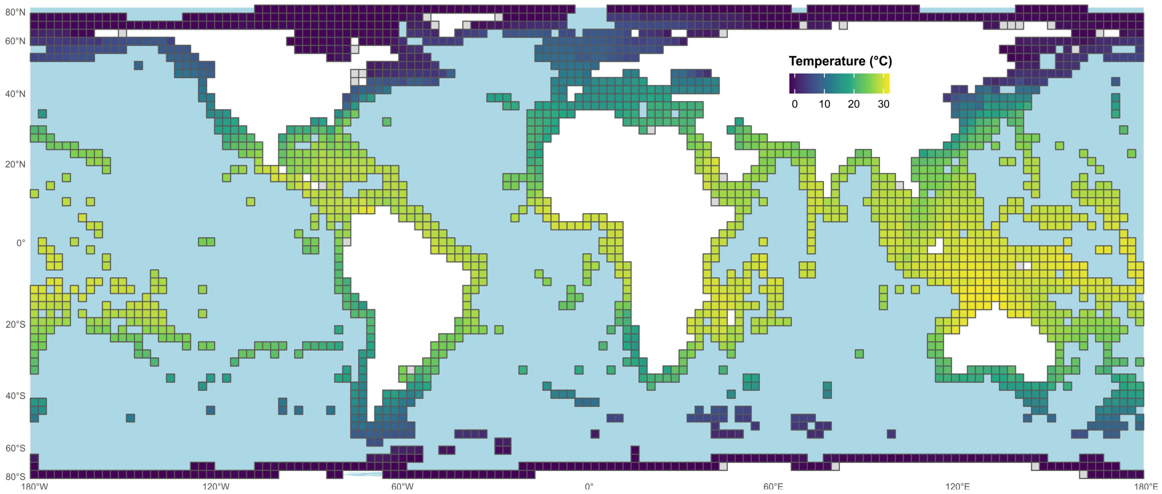


**Figure S4.** Mean global sea-surface temperatures for each grid cell used in this study. The underlying temperature data is the aggregated raster data shown in Figure S3.

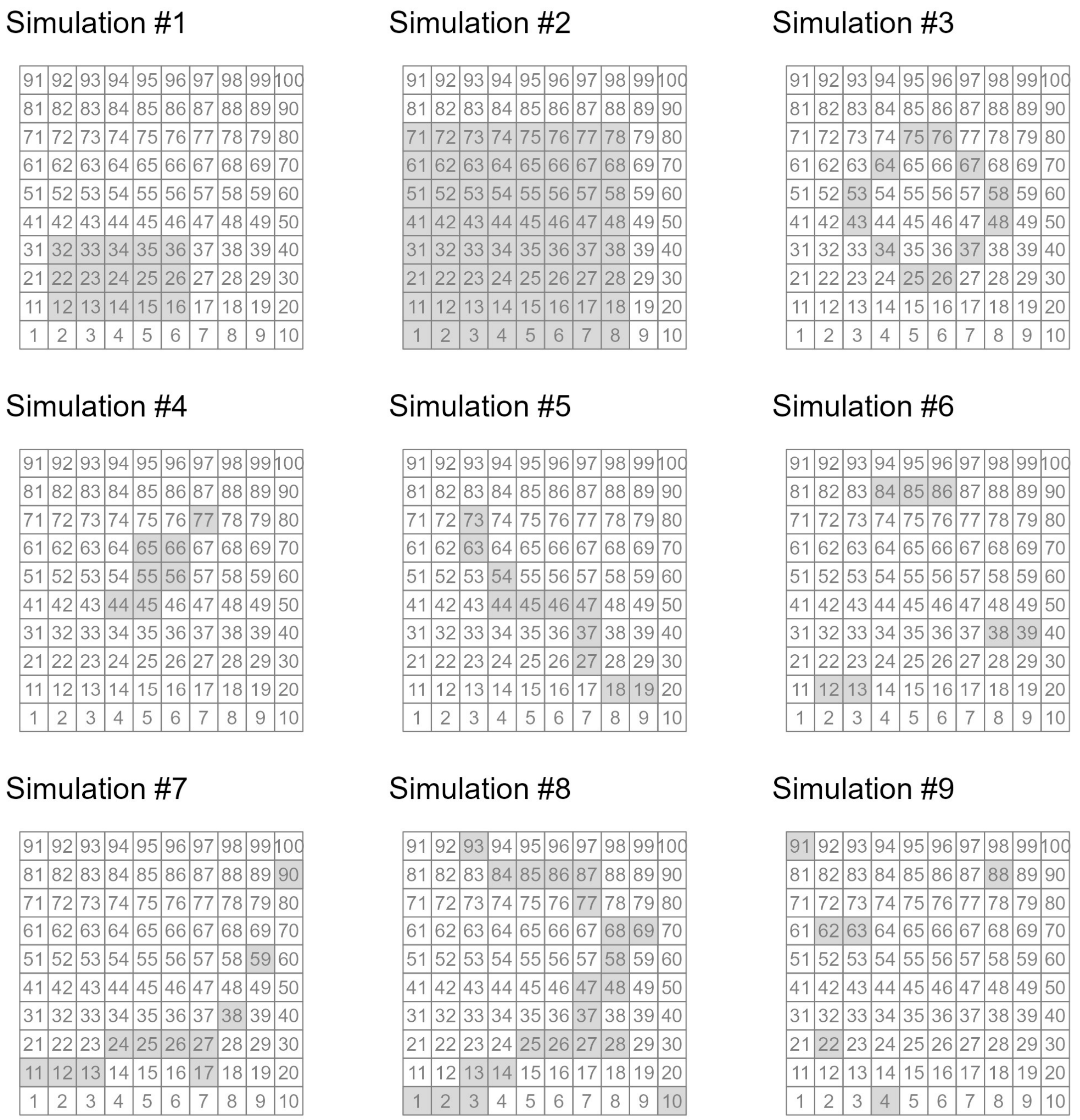


**Figure S5.** Nine hypothetical cluster arrangements for which $I_{CC}$ was computed across various cell sizes (Figure S6). Simulations were created to cover a wide range of theoretical shapes and sizes of clusters, matching biogeographic regions along coastlines (with and without outliers), within inland seas or those combining several islands.

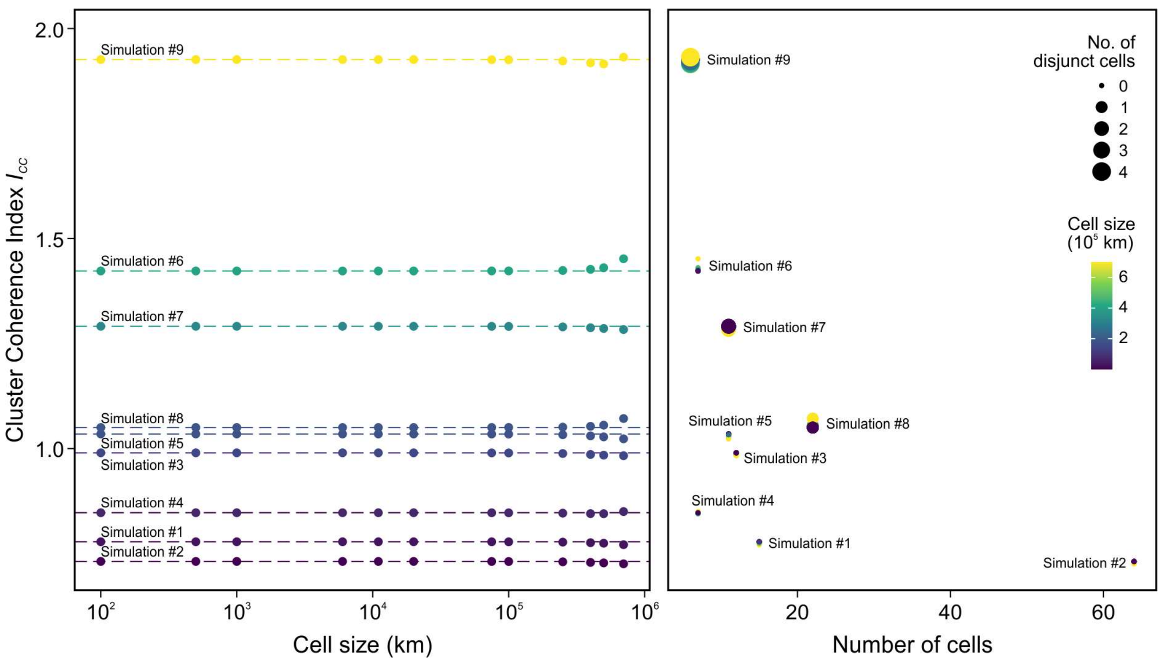


**Figure S6.** Outcome of the simulations of $I_{CC}$ for the nine hypothetical cluster arrangements (Figure S5). A. Impact of cell size on $I_{CC}$. The index remains constant for most cell sizes, only above an edge length of $10^5$ km values depart significantly. Dashed lines indicate the median values across cell sizes. B. Impact of number of cells per cluster on $I_{CC}$. A higher number of cells decreases the index, corresponding to the likelihood that a cluster is robust. In turn, a higher number of disjunct cells (i.e., single cells without neighboring cells of the same cluster) increases $I_{CC}$.

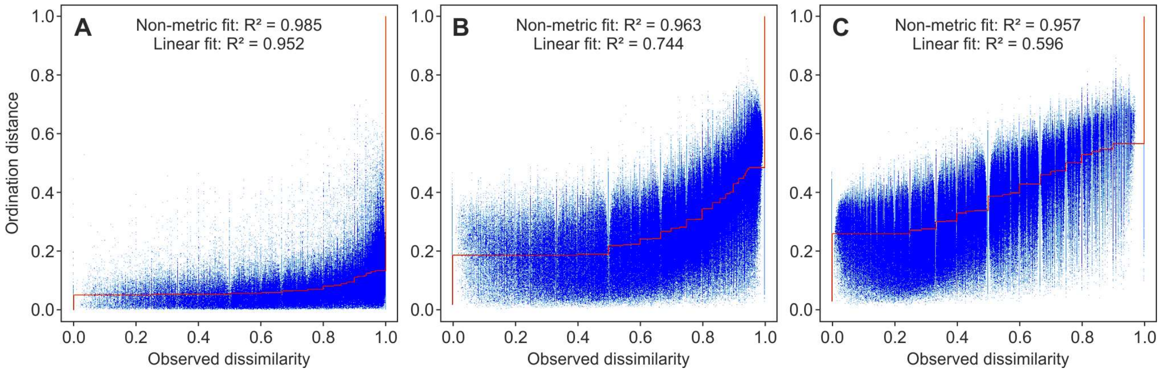


**Figure S7.** Stress plots for the non-metric Multidimensional Scalings (nMDS) of species (A), genera (B), and families (C).

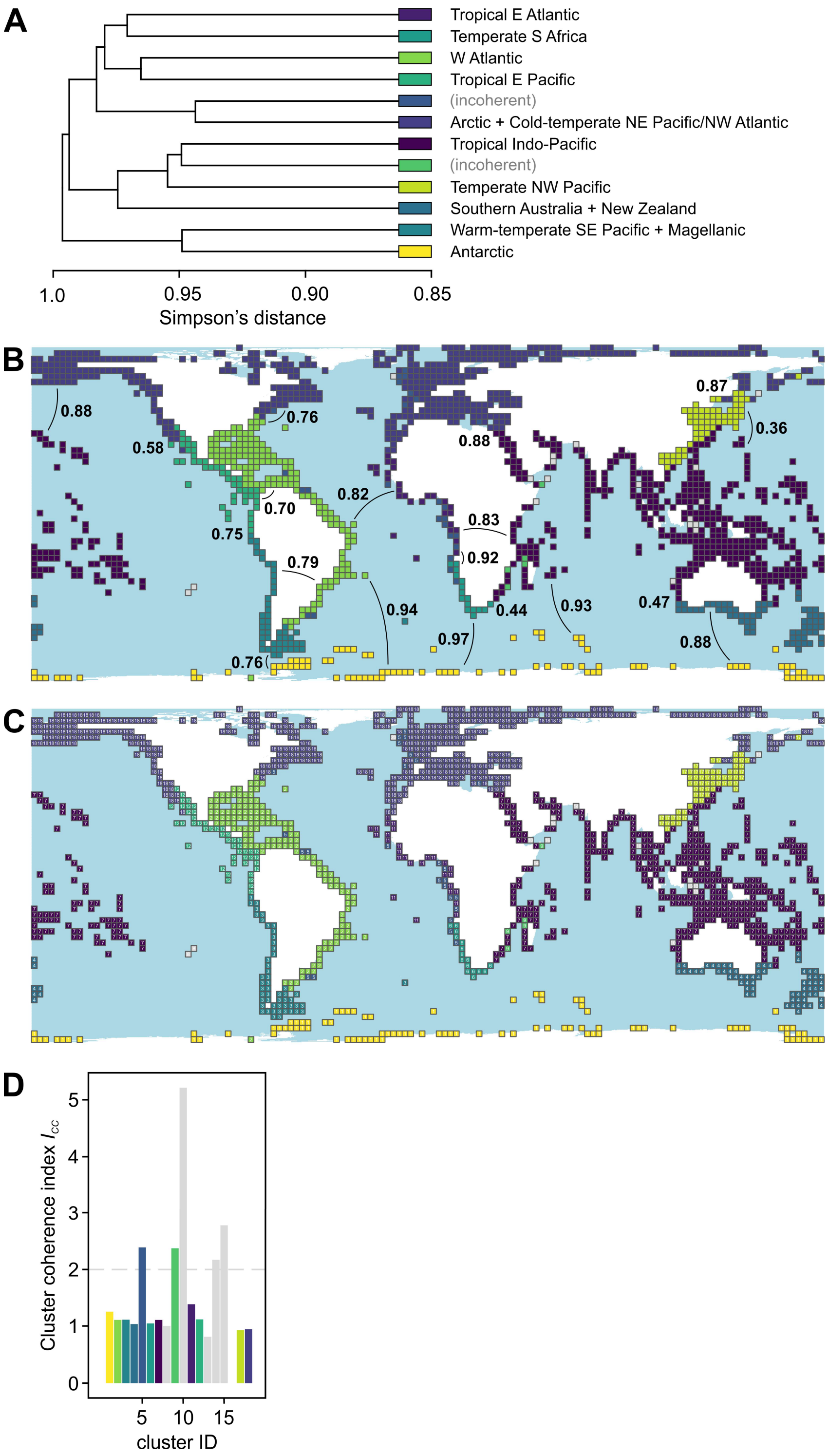


**Figure S8.** Supporting results of the cluster analyses for species-level data, at a Simpson's distance cut-off of 0.94. **A.** Simplified cluster dendrograms. Names of biogeographic units were chosen to match Spalding *et al.* [6] as closely as possible. Clusters found to be incoherent according to the Cluster Coherence Index $I_{CC}$ (D) are marked gray. **B.** Distribution of clusters on the global map. Numbers on the map refer to Simpson's distances among adjacent clusters. **C.** Cluster map as in (B) but with indication of cluster IDs, for reference to (D). **D.** Cluster Coherence Index $I_{CC}$ for each cluster. Cluster IDs as in (C); incoherent clusters marked gray. Grid and projection as in Figure 1.

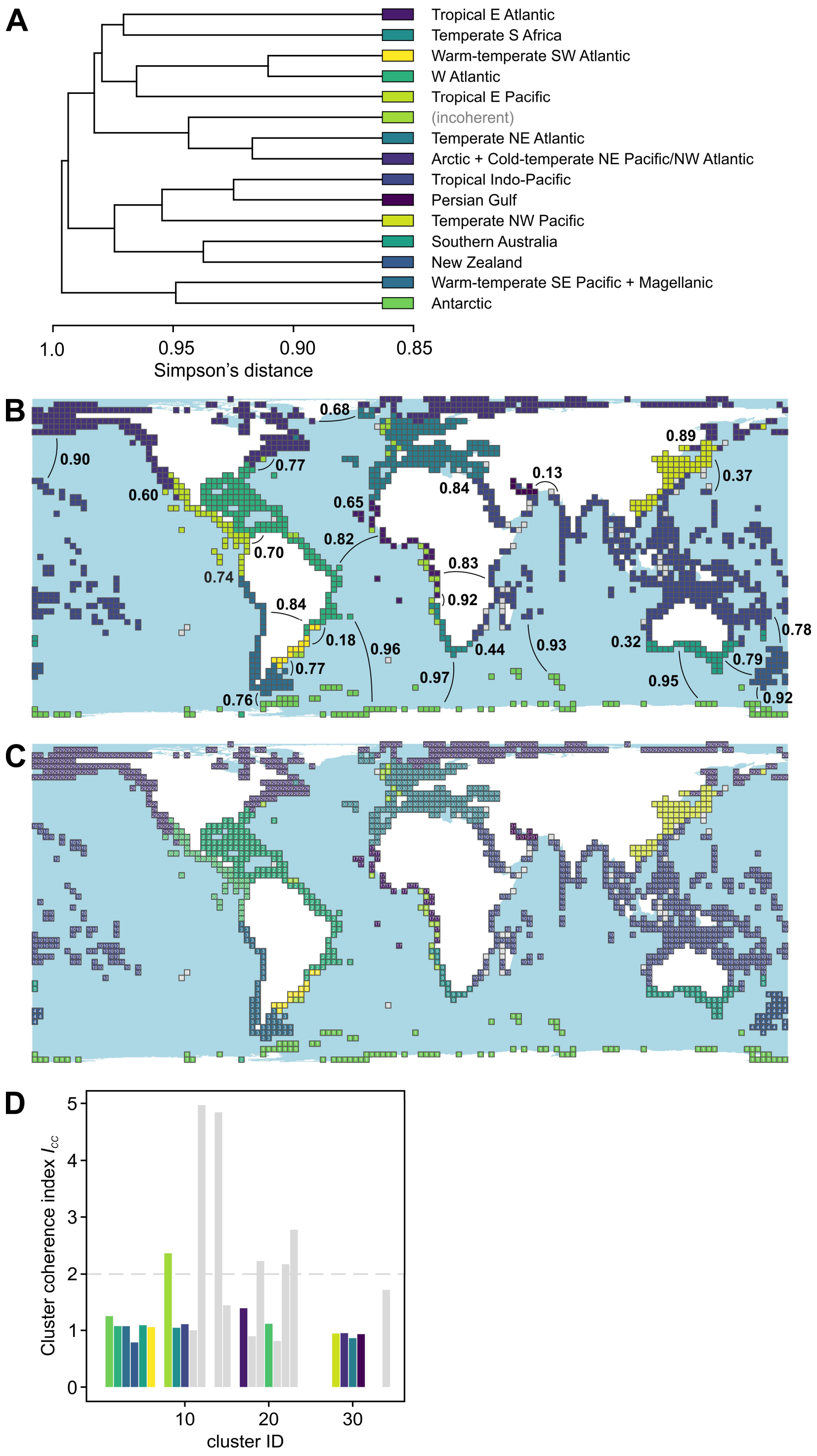


**Figure S9.** Supporting results of the cluster analyses for species-level data, at a Simpson's distance cut-off of 0.90. For details see Figure S8.

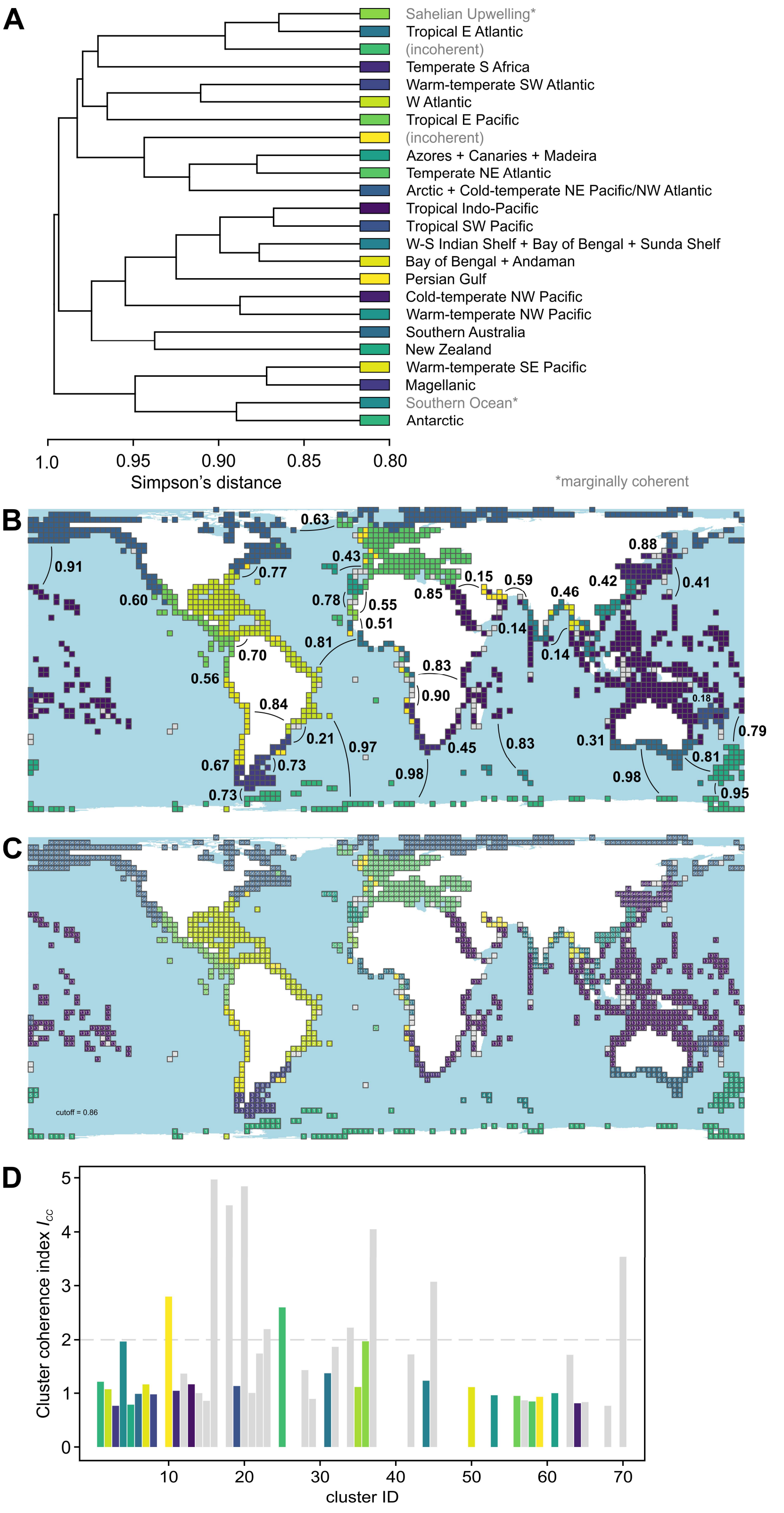


**Figure S10.** Supporting results of the cluster analyses for species-level data, at a Simpson's distance cut-off of 0.86. For details see Figure S8.

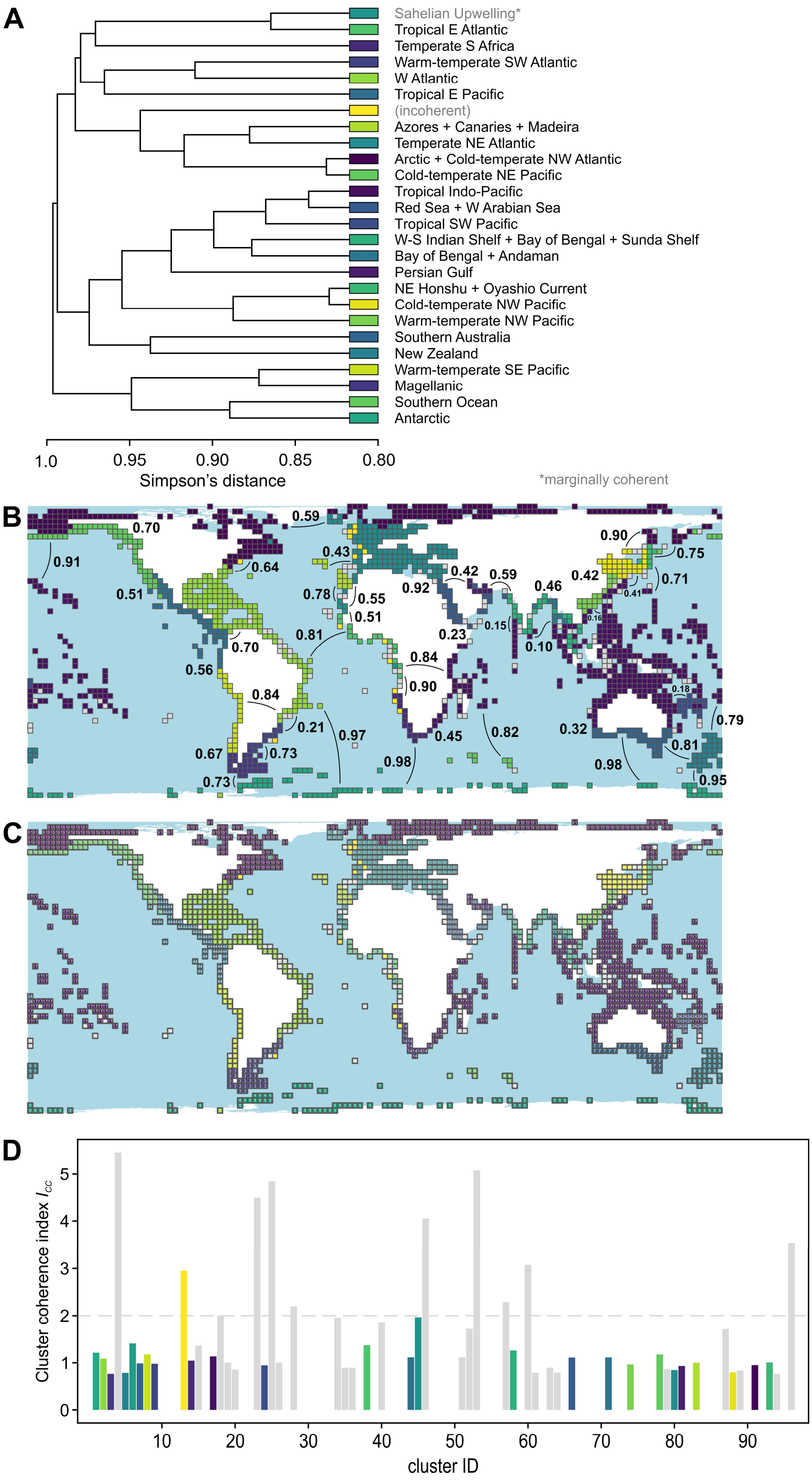


**Figure S11.** Supporting results of the cluster analyses for species-level data, at a Simpson's distance cut-off of 0.82. For details see Figure S8.

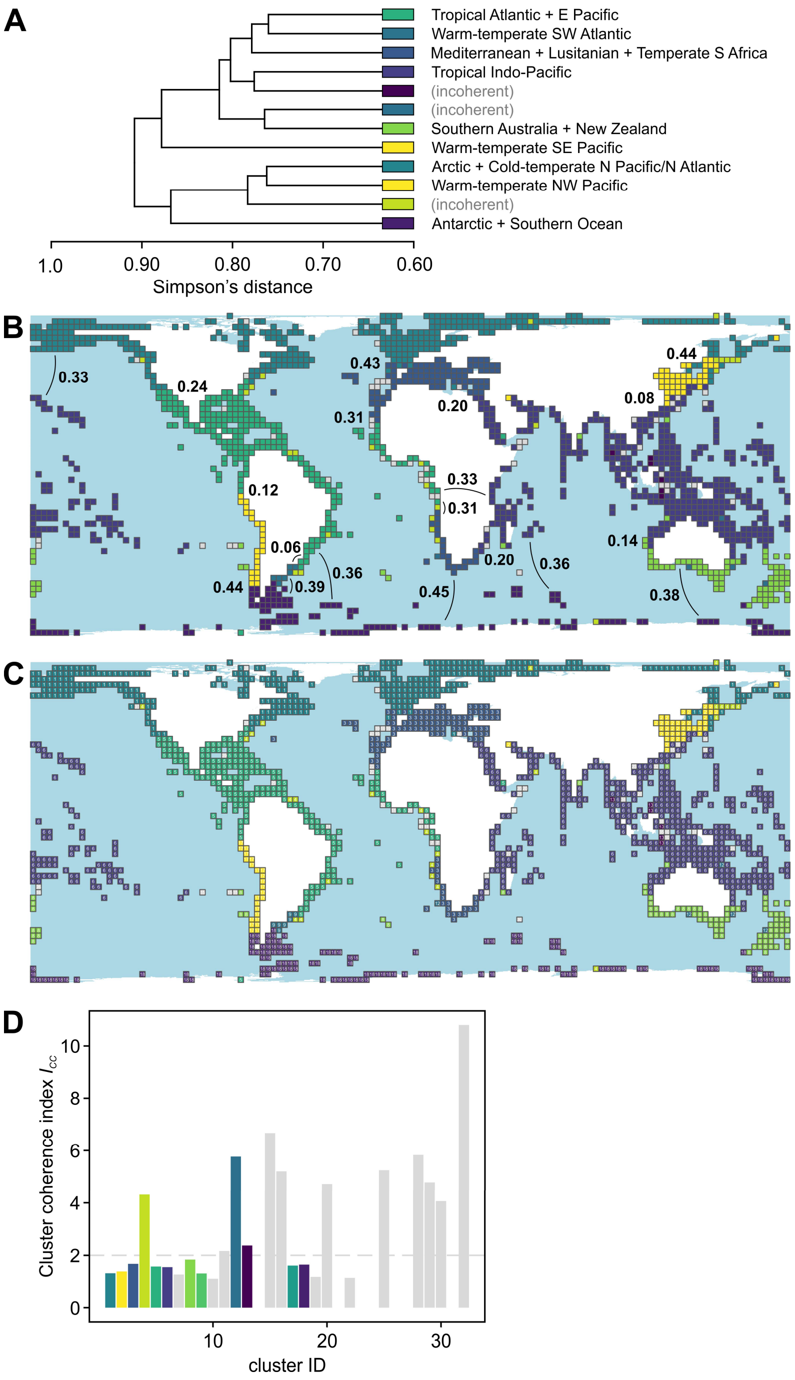


**Figure S12.** Supporting results of the cluster analyses for genus-level data, at a Simpson's distance cut-off of 0.75. For details see Figure S8.

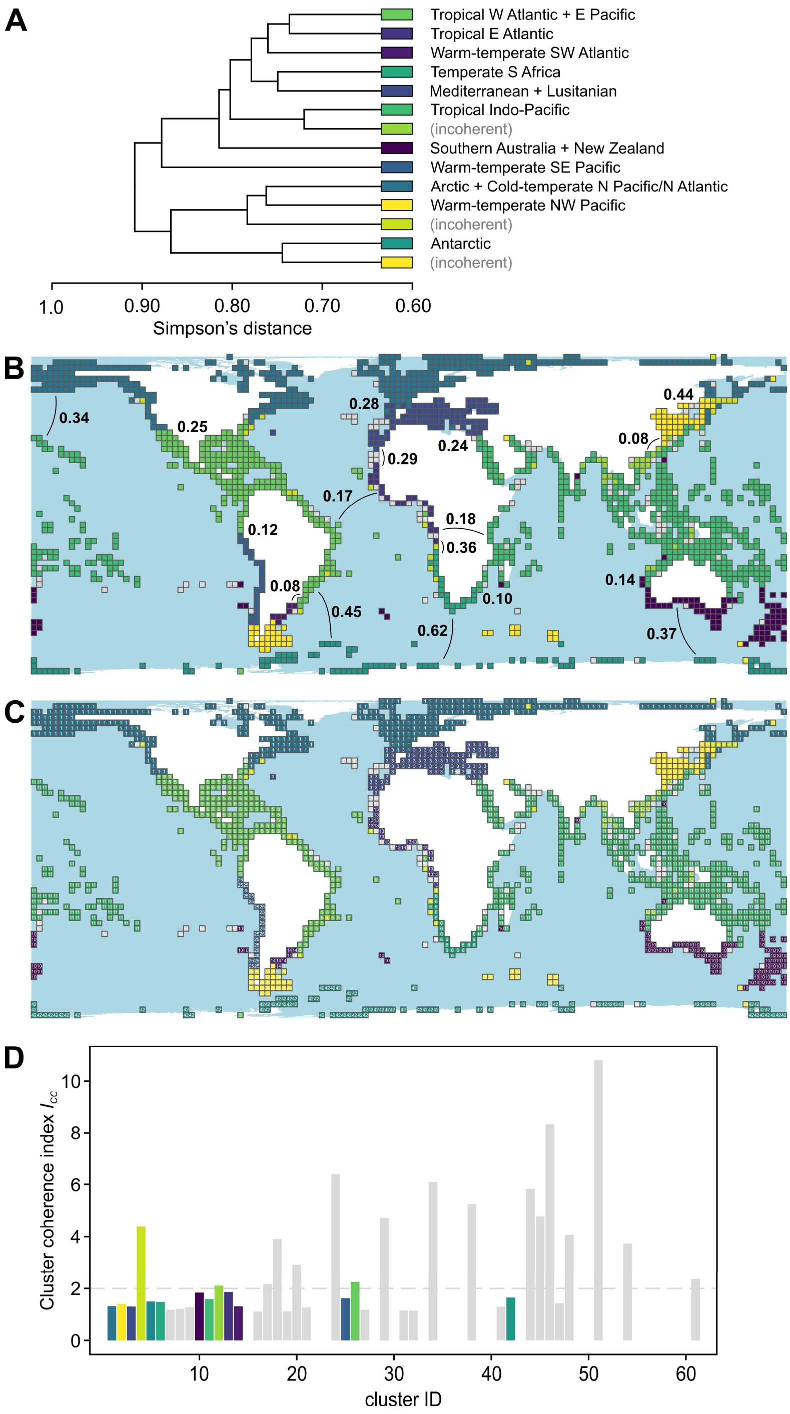


**Figure S13.** Supporting results of the cluster analyses for genus-level data, at a Simpson's distance cut-off of 0.71. For details see Figure S8.

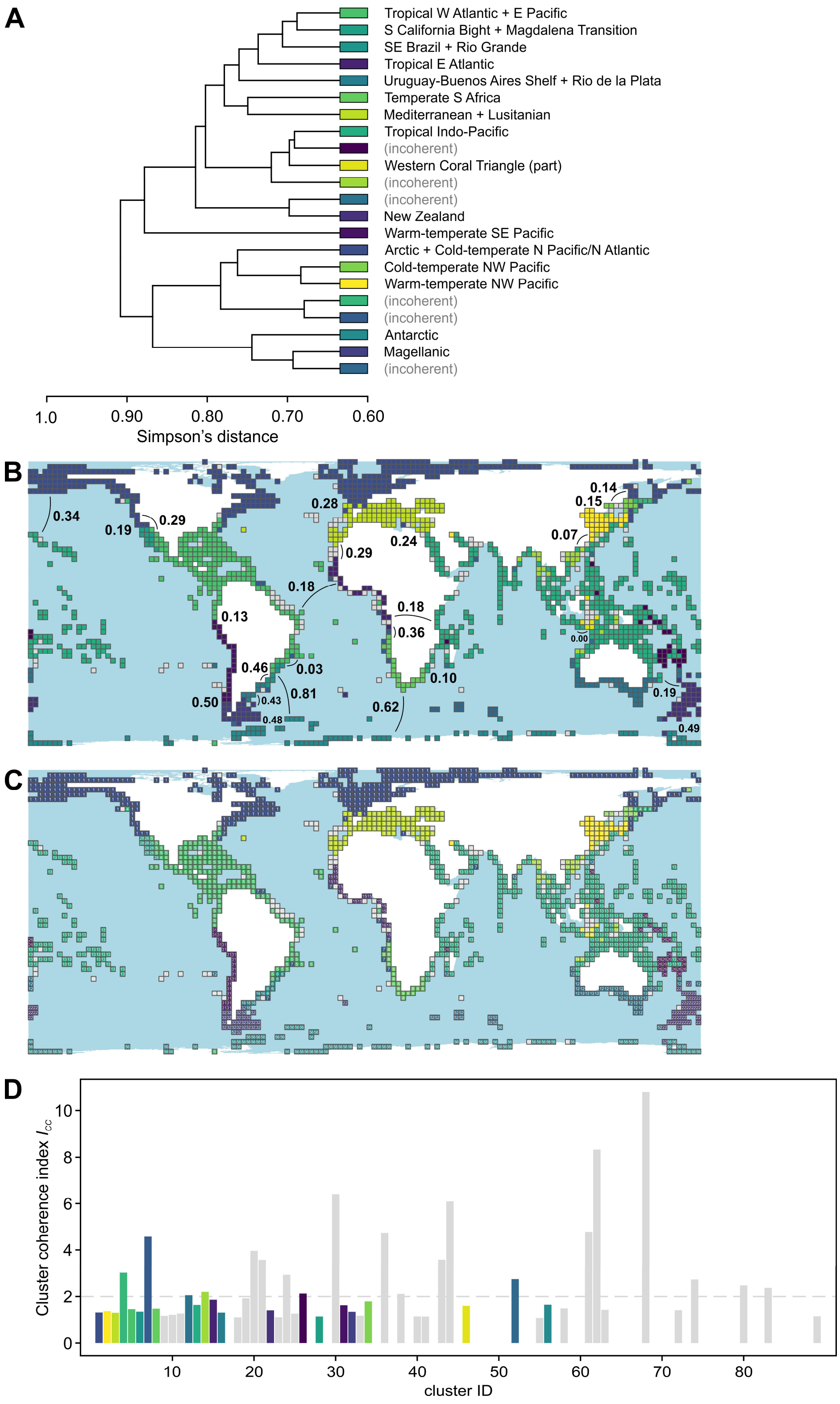


**Figure S14.** Supporting results of the cluster analyses for genus-level data, at a Simpson's distance cut-off of 0.67. For details see Figure S8.

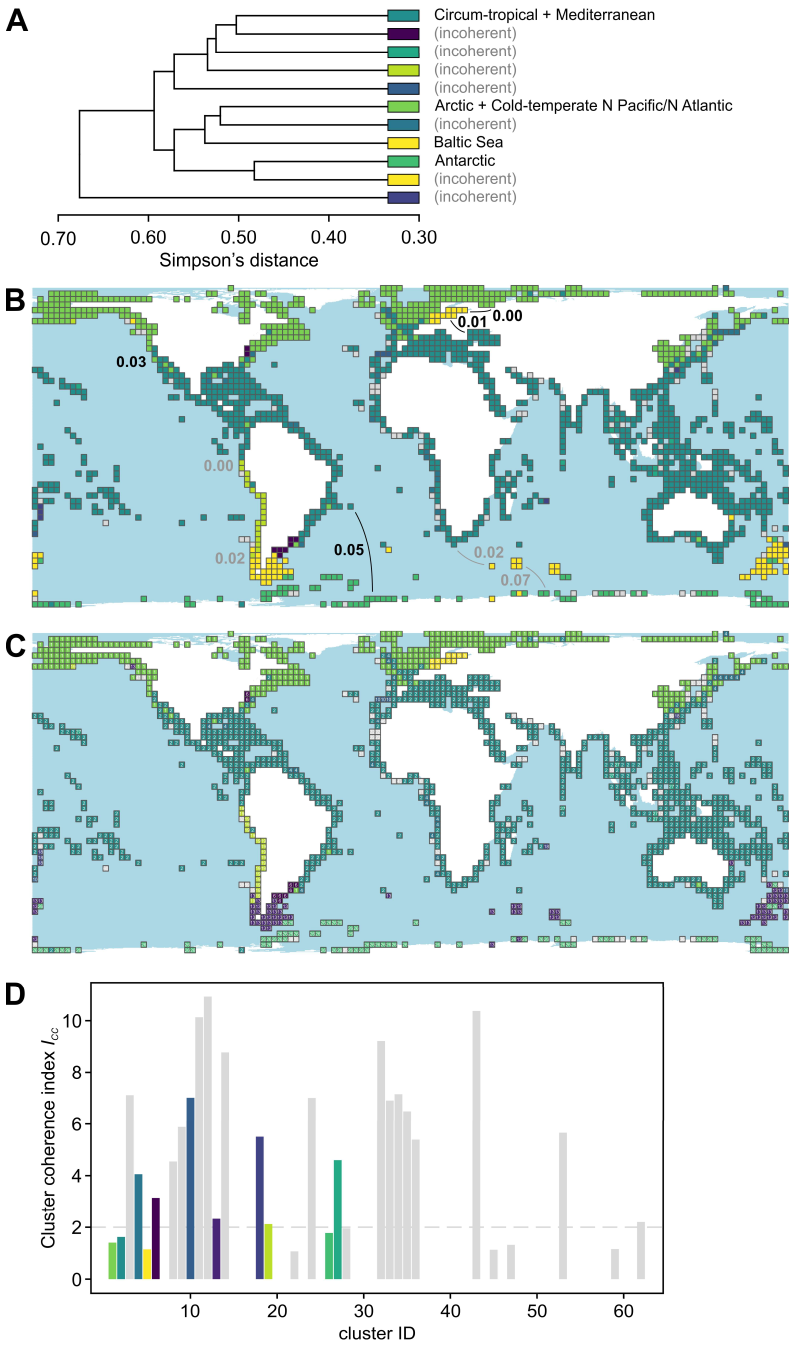


**Figure S15.** Supporting results of the cluster analyses for family-level data, at a Simpson's distance cut-off of 0.48. For details see Figure S8.

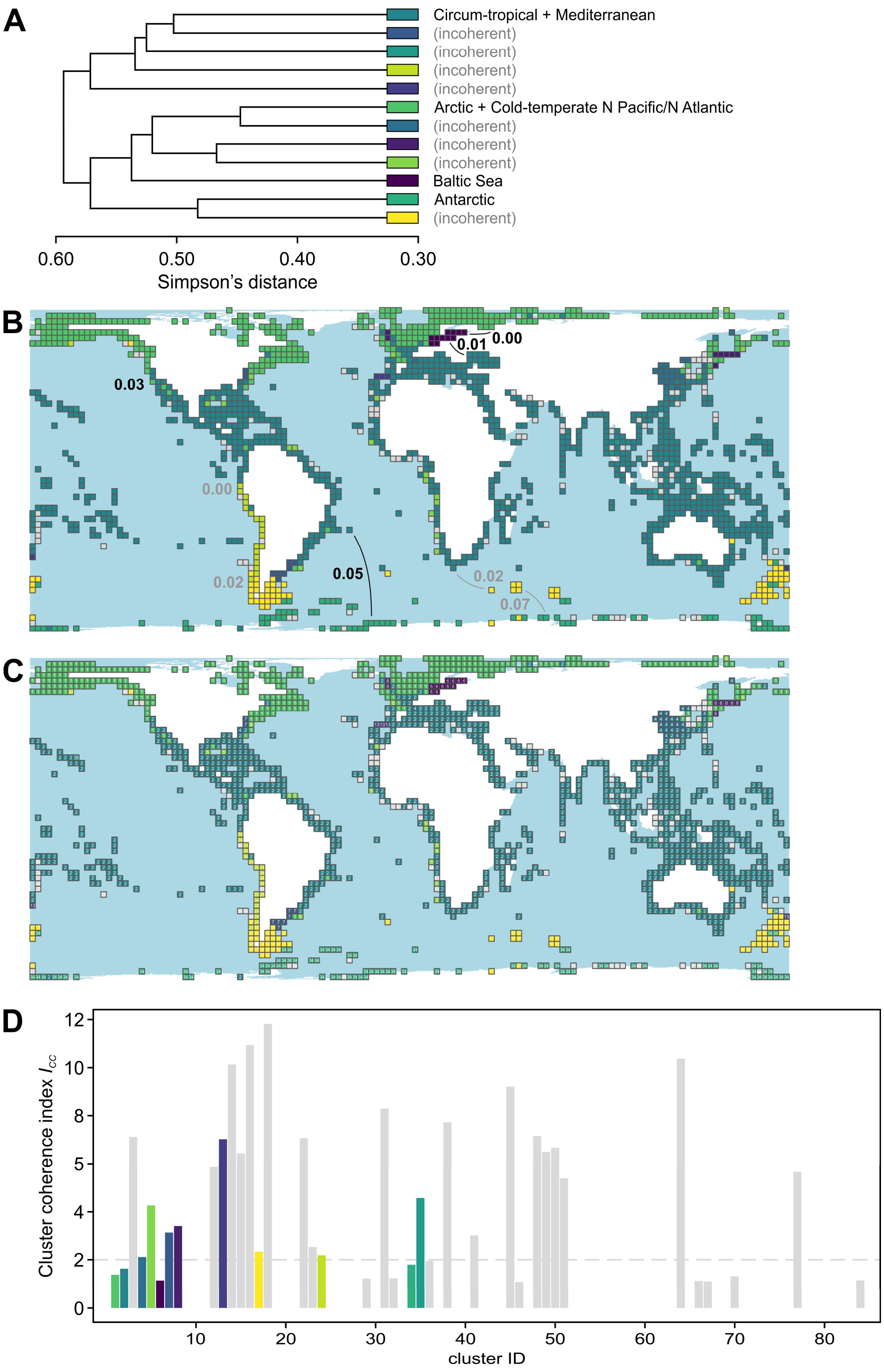


**Figure S16.** Supporting results of the cluster analyses for family-level data, at a Simpson's distance cut-off of 0.44. For details see Figure S8.

**Table S1.** Number of species and degree of endemism per biogeographic unit resulting from the cluster analysis (species data, cut-off: 0.94).

| **Biogeographic unit** | **Cluster ID** | **No. endemics** | **No. species total** | **Endemism %** |
|---|---|---|---|---|
| Antarctic | 1 | 495 | 794 | 62.3 |
| W Atlantic | 2 | 2751 | 5187 | 53.0 |
| Warm-temperate SE Pacific + Magellanic | 3 | 353 | 944 | 37.4 |
| New Zealand + Southern Australia | 4 | 2328 | 5278 | 44.1 |
| (incoherent) | 5 | 163 | 1068 | 15.3 |
| Temperate S Africa | 6 | 580 | 1598 | 36.3 |
| Tropical Indo-Pacific | 7 | 8383 | 14260 | 58.8 |
| (incoherent) | 9 | 25 | 153 | 16.3 |
| Tropical E Atlantic | 11 | 422 | 946 | 44.6 |
| Tropical E Pacific | 12 | 1382 | 4030 | 34.3 |
| Temperate NW Pacific | 17 | 1057 | 3846 | 27.5 |
| Arctic + Cold-temperate NE Pacific/NW Atlantic | 18 | 2639 | 6155 | 42.9 |

**Table S2.** Number of species and degree of endemism per biogeographic unit resulting from the cluster analysis (species data, cut-off: 0.90).

| **Biogeographic region** | **Cluster ID** | **No. endemics** | **No. species total** | **Endemism %** |
|---|---|---|---|---|
| Antarctic | 1 | 495 | 794 | 62.3 |
| W Atlantic | 2 | 2518 | 5090 | 49.5 |
| Warm-temperate SE Pacific + Magellanic | 3 | 347 | 935 | 37.1 |
| New Zealand | 4 | 1182 | 1752 | 67.5 |
| Southern Australia | 5 | 1060 | 3899 | 27.2 |
| Warm-temperate SW Atlantic | 6 | 33 | 548 | 6.0 |
| (incoherent) | 8 | 164 | 1053 | 15.6 |
| Temperate S Africa | 9 | 586 | 1598 | 36.7 |
| Tropical Indo-Pacific | 10 | 8190 | 14111 | 58.0 |
| Tropical E Atlantic | 17 | 422 | 946 | 44.6 |
| Tropical E Pacific | 20 | 1383 | 4030 | 34.3 |
| Temperate NW Pacific | 28 | 1032 | 3724 | 27.7 |
| Arctic + Cold-temperate NE Pacific/NW Atlantic | 29 | 1337 | 4371 | 30.6 |
| Temperate NE Atlantic | 30 | 1064 | 2618 | 40.6 |
| Persian Gulf | 31 | 56 | 458 | 12.2 |

**Table S3.** Number of species and degree of endemism per biogeographic unit resulting from the cluster analysis (species data, cut-off: 0.86). Asterisks mark marginally coherent clusters.

| **Biogeographic region** | **Cluster ID** | **No. endemics** | **No. species total** | **Endemism %** |
|---|---|---|---|---|
| Antarctic | 1 | 377 | 643 | 58.6 |
| W Atlantic | 2 | 2578 | 5083 | 50.7 |
| Magellanic | 3 | 181 | 604 | 30.0 |
| Southern Ocean | 4 | 67 | 265 | 25.3 |
| New Zealand | 5 | 1203 | 1752 | 68.7 |
| Southern Australia | 6 | 995 | 3642 | 27.3 |
| Warm-temperate SE Pacific | 7 | 102 | 492 | 20.7 |
| Warm-temperate SW Atlantic | 8 | 27 | 433 | 6.2 |
| (incoherent) | 10 | 145 | 947 | 15.3 |
| Temperate S Africa | 11 | 588 | 1598 | 36.8 |
| Tropical Indo-Pacific | 13 | 6202 | 13364 | 46.4 |
| Tropical SW Pacific | 19 | 388 | 2302 | 16.9 |
| (incoherent) | 25 | 148 | 304 | 48.7 |
| Tropical E Atlantic | 31 | 219 | 580 | 37.8 |
| Tropical E Pacific | 35 | 1383 | 4030 | 34.3 |
| Sahelian Upwelling* | 36 | 31 | 209 | 14.8 |
| W-S Indian Shelf + Bay of Bengal + Sunda Shelf | 44 | 166 | 1865 | 8.9 |
| Bay of Bengal + Andaman | 50 | 30 | 600 | 5.0 |
| Warm-temperate NW Pacific | 53 | 77 | 1357 | 5.7 |
| Arctic + Cold-temperate NE Pacific/NW Atlantic | 56 | 1331 | 4346 | 30.6 |
| Temperate NE Atlantic | 58 | 743 | 2227 | 33.4 |
| Persian Gulf | 59 | 56 | 458 | 12.2 |
| Azores + Canaries + Madeira | 61 | 184 | 721 | 25.5 |
| Cold-temperate NW Pacific | 64 | 868 | 3139 | 27.7 |

**Table S4.** Number of species and degree of endemism per biogeographic unit resulting from the cluster analysis (species data, cut-off: 0.82). Asterisks mark marginally coherent clusters.

| Biogeographic region | Cluster ID | No. endemics | No. species total | Endemism % |
|---|---|---|---|---|
| Antarctic | 1 | 389 | 643 | 60.5 |
| W Atlantic | 2 | 2598 | 5066 | 51.3 |
| Magellanic | 3 | 183 | 604 | 30.3 |
| New Zealand | 5 | 1218 | 1752 | 69.5 |
| Southern Ocean | 6 | 46 | 185 | 24.9 |
| Southern Australia | 7 | 997 | 3629 | 27.5 |
| Warm-temperate SE Pacific | 8 | 100 | 489 | 20.4 |
| Warm-temperate SW Atlantic | 9 | 27 | 433 | 6.2 |
| (incoherent) | 13 | 131 | 823 | 15.9 |
| Temperate S Africa | 14 | 589 | 1598 | 36.9 |
| Tropical Indo-Pacific | 17 | 5767 | 12973 | 44.5 |
| Tropical SW Pacific | 24 | 388 | 2269 | 17.1 |
| Tropical E Atlantic | 38 | 237 | 580 | 40.9 |
| Tropical E Pacific | 44 | 1384 | 4030 | 34.3 |
| Sahelian Upwelling* | 45 | 31 | 209 | 14.8 |
| W-S Indian Shelf + Bay of Bengal + Sunda Shelf | 58 | 159 | 1849 | 8.6 |
| NE Honshu + Oyashio Current | 66 | 218 | 1407 | 15.5 |
| Bay of Bengal + Andaman | 71 | 30 | 600 | 5.0 |
| Warm-temperate NW Pacific | 74 | 71 | 1333 | 5.3 |
| Cold-temperate NE Pacific | 78 | 868 | 3021 | 28.7 |
| Temperate NE Atlantic | 80 | 747 | 2227 | 33.5 |
| Persian Gulf | 81 | 57 | 458 | 12.4 |
| Azores + Canaries + Madeira | 83 | 189 | 721 | 26.2 |
| Cold-temperate NW Pacific | 88 | 710 | 3013 | 23.6 |
| Arctic + Cold-temperate NW Atlantic | 91 | 273 | 1883 | 14.5 |
| NE Honshu + Oyashio Current | 93 | 62 | 462 | 13.4 |

**Table S5.** Number of species and degree of endemism per biogeographic unit resulting from the cluster analysis (genus data, cut-off: 0.75).

| Biogeographic region | Cluster ID | No. endemics | No. species total | Endemism % |
|---|---|---|---|---|
| Arctic + Cold-temperate N Pacific/N Atlantic | 1 | 98 | 1641 | 6.0 |
| Warm-temperate NW Pacific | 2 | 14 | 1248 | 1.1 |
| Mediterranean + Lusitanian + Temperate S Africa | 3 | 96 | 1760 | 5.5 |
| (incoherent) | 4 | 10 | 701 | 1.4 |
| Tropical Atlantic + E Pacific | 5 | 272 | 2301 | 11.8 |
| Tropical Indo-Pacific | 6 | 450 | 3046 | 14.8 |
| Southern Australia + New Zealand | 8 | 144 | 2021 | 7.1 |
| Warm-temperate SW Atlantic | 9 | 0 | 195 | 0.0 |
| (incoherent) | 12 | 2 | 394 | 0.5 |
| (incoherent) | 13 | 1 | 302 | 0.3 |
| Warm-temperate SE Pacific | 17 | 7 | 364 | 1.9 |
| Antarctic + Southern Ocean | 18 | 86 | 596 | 14.4 |

**Table S6.** Number of species and degree of endemism per biogeographic unit resulting from the cluster analysis (genus data, cut-off: 0.71).

| Biogeographic region | Cluster ID | No. endemics | No. species total | Endemism % |
|---|---|---|---|---|
| Arctic + Cold-temperate N Pacific/N Atlantic | 1 | 100 | 1640 | 6.1 |
| Warm-temperate NW Pacific | 2 | 15 | 1245 | 1.2 |
| Mediterranean + Lusitanian | 3 | 45 | 1100 | 4.1 |
| (incoherent) | 4 | 10 | 665 | 1.5 |
| Tropical E Atlantic + E Pacific | 5 | 261 | 2227 | 11.7 |
| Temperate S Africa | 6 | 45 | 1196 | 3.8 |
| Southern Australia + New Zealand | 10 | 163 | 2021 | 8.1 |
| Tropical Indo-Pacific | 11 | 422 | 3015 | 14.0 |
| (incoherent) | 12 | 4 | 843 | 0.5 |
| Tropical E Atlantic | 13 | 15 | 422 | 3.6 |
| Warm-temperate SW Atlantic | 14 | 0 | 195 | 0.0 |
| Warm-temperate SE Pacific | 25 | 7 | 361 | 1.9 |
| (incoherent) | 26 | 22 | 453 | 4.9 |
| Antarctic | 42 | 41 | 342 | 12.0 |

**Table S7.** Number of species and degree of endemism per biogeographic unit resulting from the cluster analysis (genus data, cut-off: 0.67).

| Biogeographic region | Cluster ID | No. endemics | No. species total | Endemism % |
|---|---|---|---|---|
| Arctic + Cold-temperate N Pacific/N Atlantic | 1 | 101 | 1640 | 6.2 |
| Warm-temperate NW Pacific | 2 | 13 | 1195 | 1.1 |
| Mediterranean + Lusitanian | 3 | 45 | 1097 | 4.1 |
| (incoherent) | 4 | 5 | 155 | 3.2 |
| Tropical E Atlantic + E Pacific | 5 | 193 | 2133 | 9.0 |
| SE Brazil + Rio Grande | 6 | 1 | 315 | 0.3 |
| (incoherent) | 7 | 3 | 591 | 0.5 |
| Temperate S Africa | 8 | 45 | 1196 | 3.8 |
| (incoherent) | 12 | 65 | 1758 | 3.7 |
| Tropical Indo-Pacific | 13 | 321 | 2985 | 10.8 |
| (incoherent) | 14 | 4 | 821 | 0.5 |
| Tropical E Atlantic | 15 | 15 | 422 | 3.6 |
| Uruguay-Buenos Aires Shelf + Rio de la Plata | 16 | 0 | 195 | 0.0 |
| New Zealand | 22 | 74 | 960 | 7.7 |
| (incoherent) | 26 | 8 | 1012 | 0.8 |
| S California Bight + Magdalena Transition | 28 | 3 | 851 | 0.4 |
| Warm-temperate SE Pacific | 31 | 7 | 361 | 1.9 |
| Magellanic | 32 | 19 | 398 | 4.8 |
| Cold-temperate NW Pacific | 34 | 2 | 343 | 0.6 |
| Western Coral Triangle (part) | 46 | 0 | 250 | 0.0 |
| (incoherent) | 52 | 3 | 169 | 1.8 |
| Antarctic | 56 | 41 | 342 | 12.0 |

**Table S8.** Number of species and degree of endemism per biogeographic unit resulting from the cluster analysis (family data, cut-off: 0.48).

| Biogeographic region | Cluster ID | No. endemics | No. species total | Endemism % |
|---|---|---|---|---|
| Arctic + Cold-temperate N Pacific/N Atlantic | 1 | 8 | 434 | 1.8 |
| Circum-tropical + Mediterranean | 2 | 42 | 498 | 8.4 |
| (incoherent) | 4 | 0 | 266 | 0.0 |
| Baltic Sea | 5 | 0 | 132 | 0.0 |
| (incoherent) | 6 | 0 | 128 | 0.0 |
| (incoherent) | 10 | 0 | 156 | 0.0 |
| (incoherent) | 13 | 1 | 377 | 0.3 |
| (incoherent) | 18 | 0 | 126 | 0.0 |
| (incoherent) | 19 | 0 | 171 | 0.0 |
| Antarctica | 26 | 6 | 287 | 2.1 |
| (incoherent) | 27 | 0 | 148 | 0.0 |

**Table S9.** Number of species and degree of endemism per biogeographic unit resulting from the cluster analysis (family data, cut-off: 0.44).

| Biogeographic region | Cluster ID | No. endemics | No. species total | Endemism % |
|---|---|---|---|---|
| Arctic + Cold-temperate N Pacific/N Atlantic | 1 | 8 | 430 | 1.9 |
| Circum-tropical + Mediterranean | 2 | 44 | 498 | 8.8 |
| (incoherent) | 4 | 0 | 255 | 0.0 |
| (incoherent) | 5 | 0 | 246 | 0.0 |
| Baltic Sea | 6 | 0 | 132 | 0.0 |
| (incoherent) | 7 | 0 | 128 | 0.0 |
| (incoherent) | 8 | 0 | 119 | 0.0 |
| (incoherent) | 13 | 0 | 156 | 0.0 |
| (incoherent) | 17 | 1 | 377 | 0.3 |
| (incoherent) | 24 | 0 | 169 | 0.0 |
| Antarctica | 34 | 6 | 286 | 2.1 |
| (incoherent) | 35 | 0 | 145 | 0.0 |

**Table S10.** Simpson's distances among all clusters resulting from the cluster analysis of species data (cut-off: 0.94).

| Cluster ID1 | Cluster ID2 | Simpson's distance |
|---|---|---|
| 1 | 2 | 0.937 |
| 1 | 3 | 0.757 |
| 1 | 4 | 0.882 |
| 1 | 5 | 0.965 |
| 1 | 6 | 0.967 |
| 1 | 7 | 0.928 |
| 1 | 9 | 0.98 |
| 1 | 11 | 0.99 |
| 1 | 12 | 0.979 |
| 1 | 17 | 0.962 |
| 1 | 18 | 0.95 |
| 2 | 3 | 0.787 |
| 2 | 4 | 0.958 |
| 2 | 5 | 0.626 |
| 2 | 6 | 0.919 |
| 2 | 7 | 0.874 |
| 2 | 9 | 0.908 |
| 2 | 11 | 0.821 |
| 2 | 12 | 0.702 |
| 2 | 17 | 0.932 |
| 2 | 18 | 0.761 |
| 3 | 4 | 0.93 |
| 3 | 5 | 0.952 |
| 3 | 6 | 0.968 |
| 3 | 7 | 0.921 |
| 3 | 9 | 0.993 |
| 3 | 11 | 0.986 |
| 3 | 12 | 0.746 |
| 3 | 17 | 0.961 |
| 3 | 18 | 0.792 |
| 4 | 5 | 0.957 |
| 4 | 6 | 0.85 |
| 4 | 7 | 0.473 |
| 4 | 9 | 0.784 |
| 4 | 11 | 0.95 |
| 4 | 12 | 0.96 |
| 4 | 17 | 0.812 |
| 4 | 18 | 0.956 |
| 5 | 6 | 0.934 |
| 5 | 7 | 0.895 |
| 5 | 9 | 0.967 |
| 5 | 11 | 0.785 |
| 5 | 12 | 0.9 |
| 5 | 17 | 0.945 |
| 5 | 18 | 0.351 |
| 6 | 7 | 0.443 |
| 6 | 9 | 0.804 |
| 6 | 11 | 0.918 |
| 6 | 12 | 0.937 |
| 6 | 17 | 0.838 |
| 6 | 18 | 0.88 |
| 7 | 9 | 0.203 |
| 7 | 11 | 0.828 |
| 7 | 12 | 0.866 |
| 7 | 17 | 0.363 |
| 7 | 18 | 0.885 |
| 9 | 11 | 0.954 |
| 9 | 12 | 0.974 |
| 9 | 17 | 0.693 |
| 9 | 18 | 0.922 |
| 11 | 12 | 0.921 |
| 11 | 17 | 0.937 |
| 11 | 18 | 0.63 |
| 12 | 17 | 0.939 |
| 12 | 18 | 0.581 |
| 17 | 18 | 0.867 |

**Table S11**. Simpson's distances among all clusters resulting from the cluster analysis of species data (cut-off: 0.90).

| Cluster ID1 | Cluster ID2 | Simpson's distance |
|---|---|---|
| 1 | 2 | 0.962 |
| 1 | 3 | 0.757 |
| 1 | 4 | 0.917 |
| 1 | 5 | 0.946 |
| 1 | 6 | 0.953 |
| 1 | 8 | 0.966 |
| 1 | 9 | 0.967 |
| 1 | 10 | 0.928 |
| 1 | 17 | 0.99 |
| 1 | 20 | 0.979 |
| 1 | 28 | 0.965 |
| 1 | 29 | 0.957 |
| 1 | 30 | 0.966 |
| 1 | 31 | 0.989 |
| 2 | 3 | 0.842 |
| 2 | 4 | 0.956 |
| 2 | 5 | 0.954 |
| 2 | 6 | 0.177 |
| 2 | 8 | 0.632 |
| 2 | 9 | 0.922 |
| 2 | 10 | 0.874 |
| 2 | 17 | 0.822 |
| 2 | 20 | 0.703 |
| 2 | 28 | 0.932 |
| 2 | 29 | 0.771 |
| 2 | 30 | 0.805 |
| 2 | 31 | 0.919 |
| 3 | 4 | 0.944 |
| 3 | 5 | 0.968 |
| 3 | 6 | 0.772 |
| 3 | 8 | 0.953 |
| 3 | 9 | 0.97 |
| 3 | 10 | 0.921 |
| 3 | 17 | 0.986 |
| 3 | 20 | 0.743 |
| 3 | 28 | 0.96 |
| 3 | 29 | 0.807 |
| 3 | 30 | 0.947 |
| 3 | 31 | 0.998 |
| 4 | 5 | 0.787 |
| 4 | 6 | 0.964 |
| 4 | 8 | 0.977 |
| 4 | 9 | 0.955 |
| 4 | 10 | 0.785 |
| 4 | 17 | 0.979 |
| 4 | 20 | 0.969 |
| 4 | 28 | 0.929 |
| 4 | 29 | 0.969 |
| 4 | 30 | 0.963 |
| 4 | 31 | 0.956 |
| 5 | 6 | 0.971 |
| 5 | 8 | 0.97 |
| 5 | 9 | 0.86 |
| 5 | 10 | 0.315 |
| 5 | 17 | 0.956 |
| 5 | 20 | 0.963 |
| 5 | 28 | 0.819 |
| 5 | 29 | 0.974 |
| 5 | 30 | 0.947 |
| 5 | 31 | 0.736 |
| 6 | 8 | 0.894 |
| 6 | 9 | 0.962 |
| 6 | 10 | 0.892 |
| 6 | 17 | 0.951 |
| 6 | 20 | 0.792 |
| 6 | 28 | 0.958 |
| 6 | 29 | 0.812 |
| 6 | 30 | 0.892 |
| 6 | 31 | 0.996 |
| 8 | 9 | 0.934 |
| 8 | 10 | 0.9 |
| 8 | 17 | 0.785 |
| 8 | 20 | 0.9 |
| 8 | 28 | 0.946 |
| 8 | 29 | 0.537 |
| 8 | 30 | 0.464 |
| 8 | 31 | 0.991 |
| 9 | 10 | 0.445 |
| 9 | 17 | 0.918 |
| 9 | 20 | 0.937 |
| 9 | 28 | 0.842 |
| 9 | 29 | 0.936 |
| 9 | 30 | 0.897 |
| 9 | 31 | 0.852 |
| 10 | 17 | 0.83 |
| 10 | 20 | 0.867 |
| 10 | 28 | 0.368 |
| 10 | 29 | 0.904 |
| 10 | 30 | 0.843 |
| 10 | 31 | 0.131 |
| 17 | 20 | 0.921 |
| 17 | 28 | 0.939 |
| 17 | 29 | 0.864 |
| 17 | 30 | 0.654 |
| 17 | 31 | 0.987 |
| 20 | 28 | 0.938 |
| 20 | 29 | 0.6 |
| 20 | 30 | 0.93 |
| 20 | 31 | 0.941 |
| 28 | 29 | 0.89 |
| 28 | 30 | 0.922 |
| 28 | 31 | 0.651 |
| 29 | 30 | 0.679 |
| 29 | 31 | 0.976 |
| 30 | 31 | 0.891 |

**Table S12.** Simpson's distances among all clusters resulting from the cluster analysis of species data (cut-off: 0.86).

| Cluster ID1 | Cluster ID2 | Simpson's distance |
|---|---|---|
| 1 | 2 | 0.97 |
| 1 | 3 | 0.73 |
| 1 | 4 | 0.57 |
| 1 | 5 | 0.95 |
| 1 | 6 | 0.98 |
| 1 | 7 | 0.921 |
| 1 | 8 | 0.956 |
| 1 | 10 | 0.963 |
| 1 | 11 | 0.984 |
| 1 | 13 | 0.981 |
| 1 | 19 | 0.998 |
| 1 | 25 | 1 |
| 1 | 31 | 0.99 |
| 1 | 35 | 0.986 |
| 1 | 36 | 0.986 |
| 1 | 44 | 1 |
| 1 | 50 | 0.998 |
| 1 | 53 | 0.997 |
| 1 | 56 | 0.958 |
| 1 | 58 | 0.969 |
| 1 | 59 | 1 |
| 1 | 61 | 0.988 |
| 1 | 64 | 0.991 |
| 2 | 3 | 0.853 |
| 2 | 4 | 0.943 |
| 2 | 5 | 0.956 |
| 2 | 6 | 0.953 |
| 2 | 7 | 0.837 |
| 2 | 8 | 0.21 |
| 2 | 10 | 0.607 |
| 2 | 11 | 0.922 |
| 2 | 13 | 0.882 |
| 2 | 19 | 0.943 |
| 2 | 25 | 0.803 |
| 2 | 31 | 0.812 |
| 2 | 35 | 0.704 |
| 2 | 36 | 0.775 |
| 2 | 44 | 0.888 |
| 2 | 50 | 0.9 |
| 2 | 53 | 0.91 |
| 2 | 56 | 0.77 |
| 2 | 58 | 0.802 |
| 2 | 59 | 0.919 |
| 2 | 61 | 0.727 |
| 2 | 64 | 0.929 |
| 3 | 4 | 0.713 |
| 3 | 5 | 0.927 |
| 3 | 6 | 0.97 |
| 3 | 7 | 0.673 |
| 3 | 8 | 0.727 |
| 3 | 10 | 0.947 |
| 3 | 11 | 0.969 |
| 3 | 13 | 0.955 |
| 3 | 19 | 0.998 |
| 3 | 25 | 0.997 |
| 3 | 31 | 0.993 |
| 3 | 35 | 0.907 |
| 3 | 36 | 0.99 |
| 3 | 44 | 0.99 |
| 3 | 50 | 0.998 |
| 3 | 53 | 0.988 |
| 3 | 56 | 0.914 |
| 3 | 58 | 0.95 |
| 3 | 59 | 1 |
| 3 | 61 | 0.985 |
| 3 | 64 | 0.974 |
| 4 | 5 | 0.819 |
| 4 | 6 | 0.891 |
| 4 | 7 | 0.921 |
| 4 | 8 | 0.958 |
| 4 | 10 | 0.958 |
| 4 | 11 | 0.921 |
| 4 | 13 | 0.834 |
| 4 | 19 | 0.898 |
| 4 | 25 | 0.996 |
| 4 | 31 | 0.996 |
| 4 | 35 | 0.966 |
| 4 | 36 | 0.995 |
| 4 | 44 | 0.906 |
| 4 | 50 | 0.97 |
| 4 | 53 | 0.947 |
| 4 | 56 | 0.951 |
| 4 | 58 | 0.966 |
| 4 | 59 | 0.981 |
| 4 | 61 | 0.985 |
| 4 | 64 | 0.921 |
| 5 | 6 | 0.811 |
| 5 | 7 | 0.945 |
| 5 | 8 | 0.956 |
| 5 | 10 | 0.977 |
| 5 | 11 | 0.955 |
| 5 | 13 | 0.793 |
| 5 | 19 | 0.941 |
| 5 | 25 | 0.977 |
| 5 | 31 | 0.978 |
| 5 | 35 | 0.969 |
| 5 | 36 | 0.971 |
| 5 | 44 | 0.956 |
| 5 | 50 | 0.957 |
| 5 | 53 | 0.957 |
| 5 | 56 | 0.969 |
| 5 | 58 | 0.967 |
| 5 | 59 | 0.956 |
| 5 | 61 | 0.965 |
| 5 | 64 | 0.934 |
| 6 | 7 | 0.965 |
| 6 | 8 | 0.965 |
| 6 | 10 | 0.971 |
| 6 | 11 | 0.869 |
| 6 | 13 | 0.314 |
| 6 | 19 | 0.801 |
| 6 | 25 | 0.951 |
| 6 | 31 | 0.962 |
| 6 | 35 | 0.963 |
| 6 | 36 | 0.967 |
| 6 | 44 | 0.727 |

| | | |
|---|---|---|
| 6 | 50 | 0.727 |
| 6 | 53 | 0.726 |
| 6 | 56 | 0.973 |
| 6 | 58 | 0.947 |
| 6 | 59 | 0.749 |
| 6 | 61 | 0.947 |
| 6 | 64 | 0.84 |
| 7 | 8 | 0.905 |
| 7 | 10 | 0.947 |
| 7 | 11 | 0.957 |
| 7 | 13 | 0.9 |
| 7 | 19 | 0.992 |
| 7 | 25 | 0.984 |
| 7 | 31 | 0.99 |
| 7 | 35 | 0.561 |
| 7 | 36 | 0.986 |
| 7 | 44 | 0.97 |
| 7 | 50 | 0.996 |
| 7 | 53 | 0.99 |
| 7 | 56 | 0.695 |
| 7 | 58 | 0.933 |
| 7 | 59 | 0.998 |
| 7 | 61 | 0.984 |
| 7 | 64 | 0.953 |
| 8 | 10 | 0.898 |
| 8 | 11 | 0.956 |
| 8 | 13 | 0.894 |
| 8 | 19 | 0.988 |
| 8 | 25 | 0.974 |
| 8 | 31 | 0.963 |
| 8 | 35 | 0.794 |
| 8 | 36 | 0.957 |
| 8 | 44 | 0.961 |
| 8 | 50 | 0.991 |
| 8 | 53 | 0.984 |
| 8 | 56 | 0.813 |
| 8 | 58 | 0.905 |
| 8 | 59 | 0.998 |
| 8 | 61 | 0.958 |
| 8 | 64 | 0.958 |
| 10 | 11 | 0.939 |
| 10 | 13 | 0.916 |
| 10 | 19 | 0.995 |
| 10 | 25 | 0.924 |
| 10 | 31 | 0.831 |
| 10 | 35 | 0.902 |
| 10 | 36 | 0.742 |
| 10 | 44 | 0.977 |
| 10 | 50 | 0.998 |
| 10 | 53 | 0.989 |
| 10 | 56 | 0.49 |
| 10 | 58 | 0.482 |
| 10 | 59 | 0.996 |
| 10 | 61 | 0.724 |
| 10 | 64 | 0.946 |
| 11 | 13 | 0.448 |
| 11 | 19 | 0.897 |
| 11 | 25 | 0.914 |
| 11 | 31 | 0.903 |
| 11 | 35 | 0.937 |
| 11 | 36 | 0.9 |
| 11 | 44 | 0.873 |
| 11 | 50 | 0.868 |
| 11 | 53 | 0.895 |
| 11 | 56 | 0.936 |
| 11 | 58 | 0.907 |
| 11 | 59 | 0.852 |
| 11 | 61 | 0.918 |
| 11 | 64 | 0.859 |
| 13 | 19 | 0.178 |
| 13 | 25 | 0.842 |
| 13 | 31 | 0.829 |
| 13 | 35 | 0.876 |
| 13 | 36 | 0.871 |
| 13 | 44 | 0.139 |
| 13 | 50 | 0.093 |
| 13 | 53 | 0.164 |
| 13 | 56 | 0.913 |
| 13 | 58 | 0.846 |
| 13 | 59 | 0.148 |
| 13 | 61 | 0.854 |
| 13 | 64 | 0.414 |
| 19 | 25 | 0.961 |
| 19 | 31 | 0.978 |
| 19 | 35 | 0.95 |
| 19 | 36 | 0.976 |
| 19 | 44 | 0.749 |
| 19 | 50 | 0.698 |
| 19 | 53 | 0.753 |
| 19 | 56 | 0.985 |
| 19 | 58 | 0.975 |
| 19 | 59 | 0.773 |
| 19 | 61 | 0.969 |
| 19 | 64 | 0.774 |
| 25 | 31 | 0.711 |
| 25 | 35 | 0.901 |
| 25 | 36 | 0.9 |
| 25 | 44 | 0.951 |
| 25 | 50 | 0.99 |
| 25 | 53 | 0.977 |
| 25 | 56 | 0.914 |
| 25 | 58 | 0.776 |
| 25 | 59 | 0.99 |
| 25 | 61 | 0.763 |
| 25 | 64 | 0.951 |
| 31 | 35 | 0.914 |
| 31 | 36 | 0.512 |
| 31 | 44 | 0.941 |
| 31 | 50 | 0.979 |
| 31 | 53 | 0.972 |
| 31 | 56 | 0.876 |
| 31 | 58 | 0.733 |
| 31 | 59 | 0.991 |
| 31 | 61 | 0.817 |
| 31 | 64 | 0.945 |
| 35 | 36 | 0.919 |
| 35 | 44 | 0.916 |
| 35 | 50 | 0.913 |

| | | |
|---|---|---|
| 35 | 53 | 0.934 |
| 35 | 56 | 0.6 |
| 35 | 58 | 0.929 |
| 35 | 59 | 0.941 |
| 35 | 61 | 0.921 |
| 35 | 64 | 0.933 |
| 36 | 44 | 0.957 |
| 36 | 50 | 0.976 |
| 36 | 53 | 0.981 |
| 36 | 56 | 0.756 |
| 36 | 58 | 0.55 |
| 36 | 59 | 0.995 |
| 36 | 61 | 0.78 |
| 36 | 64 | 0.957 |
| 44 | 50 | 0.458 |
| 44 | 53 | 0.565 |
| 44 | 56 | 0.943 |
| 44 | 58 | 0.925 |
| 44 | 59 | 0.59 |
| 44 | 61 | 0.949 |
| 44 | 64 | 0.686 |

| | | |
|---|---|---|
| 50 | 53 | 0.627 |
| 50 | 56 | 0.96 |
| 50 | 58 | 0.948 |
| 50 | 59 | 0.871 |
| 50 | 61 | 0.988 |
| 50 | 64 | 0.635 |
| 53 | 56 | 0.951 |
| 53 | 58 | 0.937 |
| 53 | 59 | 0.729 |
| 53 | 61 | 0.978 |
| 53 | 64 | 0.421 |
| 56 | 58 | 0.632 |
| 56 | 59 | 0.976 |
| 56 | 61 | 0.759 |
| 56 | 64 | 0.88 |
| 58 | 59 | 0.9 |
| 58 | 61 | 0.427 |
| 58 | 64 | 0.922 |
| 59 | 61 | 0.987 |
| 59 | 64 | 0.749 |
| 61 | 64 | 0.942 |

**Table S13.** Simpson's distances among all clusters resulting from the cluster analysis of species data (cut-off: 0.82).

| Cluster ID1 | Cluster ID2 | Simpson's distance |
|---|---|---|
| 1 | 2 | 0.972 |
| 1 | 3 | 0.73 |
| 1 | 5 | 0.95 |
| 1 | 6 | 0.508 |
| 1 | 7 | 0.98 |
| 1 | 8 | 0.92 |
| 1 | 9 | 0.956 |
| 1 | 13 | 0.98 |
| 1 | 14 | 0.984 |
| 1 | 17 | 0.981 |
| 1 | 24 | 0.998 |
| 1 | 38 | 0.99 |
| 1 | 44 | 0.986 |
| 1 | 45 | 0.986 |
| 1 | 58 | 1 |
| 1 | 66 | 1 |
| 1 | 71 | 0.998 |
| 1 | 74 | 0.997 |
| 1 | 78 | 0.969 |
| 1 | 80 | 0.969 |
| 1 | 81 | 1 |
| 1 | 83 | 0.988 |
| 1 | 88 | 0.991 |
| 1 | 91 | 0.972 |
| 1 | 93 | 0.996 |
| 2 | 3 | 0.853 |
| 2 | 5 | 0.956 |
| 2 | 6 | 0.93 |
| 2 | 7 | 0.953 |
| 2 | 8 | 0.836 |
| 2 | 9 | 0.21 |
| 2 | 13 | 0.665 |
| 2 | 14 | 0.922 |
| 2 | 17 | 0.885 |
| 2 | 24 | 0.942 |
| 2 | 38 | 0.812 |
| 2 | 44 | 0.704 |
| 2 | 45 | 0.775 |
| 2 | 58 | 0.888 |
| 2 | 66 | 0.9 |
| 2 | 71 | 0.9 |
| 2 | 74 | 0.909 |
| 2 | 78 | 0.838 |
| 2 | 80 | 0.802 |
| 2 | 81 | 0.919 |
| 2 | 83 | 0.727 |
| 2 | 88 | 0.93 |
| 2 | 91 | 0.643 |
| 2 | 93 | 0.937 |
| 3 | 5 | 0.927 |
| 3 | 6 | 0.67 |
| 3 | 7 | 0.97 |
| 3 | 8 | 0.671 |
| 3 | 9 | 0.727 |
| 3 | 13 | 0.982 |
| 3 | 14 | 0.969 |
| 3 | 17 | 0.957 |
| 3 | 24 | 0.998 |
| 3 | 38 | 0.993 |
| 3 | 44 | 0.907 |
| 3 | 45 | 0.99 |
| 3 | 58 | 0.992 |
| 3 | 66 | 0.997 |
| 3 | 71 | 0.998 |
| 3 | 74 | 0.988 |
| 3 | 78 | 0.937 |
| 3 | 80 | 0.95 |
| 3 | 81 | 1 |
| 3 | 83 | 0.985 |
| 3 | 88 | 0.977 |
| 3 | 91 | 0.96 |
| 3 | 93 | 0.987 |
| 5 | 6 | 0.919 |
| 5 | 7 | 0.811 |
| 5 | 8 | 0.945 |
| 5 | 9 | 0.956 |
| 5 | 13 | 0.976 |
| 5 | 14 | 0.955 |
| 5 | 17 | 0.794 |
| 5 | 24 | 0.941 |
| 5 | 38 | 0.978 |
| 5 | 44 | 0.969 |
| 5 | 45 | 0.971 |
| 5 | 58 | 0.957 |
| 5 | 66 | 0.962 |
| 5 | 71 | 0.957 |
| 5 | 74 | 0.956 |
| 5 | 78 | 0.975 |
| 5 | 80 | 0.967 |
| 5 | 81 | 0.956 |
| 5 | 83 | 0.965 |
| 5 | 88 | 0.935 |
| 5 | 91 | 0.984 |
| 5 | 93 | 0.985 |
| 6 | 7 | 0.93 |
| 6 | 8 | 0.914 |
| 6 | 9 | 0.957 |
| 6 | 13 | 0.984 |
| 6 | 14 | 0.908 |
| 6 | 17 | 0.822 |
| 6 | 24 | 0.87 |
| 6 | 38 | 1 |
| 6 | 44 | 0.957 |
| 6 | 45 | 1 |
| 6 | 58 | 0.886 |
| 6 | 66 | 0.892 |
| 6 | 71 | 0.968 |
| 6 | 74 | 0.93 |
| 6 | 78 | 0.978 |
| 6 | 80 | 0.978 |
| 6 | 81 | 0.973 |
| 6 | 83 | 0.989 |

| | | |
|---|---|---|
| 6 | 88 | 0.897 |
| 6 | 91 | 0.973 |
| 6 | 93 | 0.978 |
| 7 | 8 | 0.965 |
| 7 | 9 | 0.965 |
| 7 | 13 | 0.968 |
| 7 | 14 | 0.869 |
| 7 | 17 | 0.316 |
| 7 | 24 | 0.802 |
| 7 | 38 | 0.962 |
| 7 | 44 | 0.963 |
| 7 | 45 | 0.967 |
| 7 | 58 | 0.727 |
| 7 | 66 | 0.753 |
| 7 | 71 | 0.727 |
| 7 | 74 | 0.725 |
| 7 | 78 | 0.977 |
| 7 | 80 | 0.947 |
| 7 | 81 | 0.749 |
| 7 | 83 | 0.947 |
| 7 | 88 | 0.836 |
| 7 | 91 | 0.975 |
| 7 | 93 | 0.959 |
| 8 | 9 | 0.905 |
| 8 | 13 | 0.965 |
| 8 | 14 | 0.957 |
| 8 | 17 | 0.904 |
| 8 | 24 | 0.992 |
| 8 | 38 | 0.99 |
| 8 | 44 | 0.56 |
| 8 | 45 | 0.986 |
| 8 | 58 | 0.971 |
| 8 | 66 | 0.994 |
| 8 | 71 | 0.996 |
| 8 | 74 | 0.99 |
| 8 | 78 | 0.706 |
| 8 | 80 | 0.933 |
| 8 | 81 | 0.998 |
| 8 | 83 | 0.984 |
| 8 | 88 | 0.953 |
| 8 | 91 | 0.943 |
| 8 | 93 | 0.989 |
| 9 | 13 | 0.963 |
| 9 | 14 | 0.956 |
| 9 | 17 | 0.896 |
| 9 | 24 | 0.988 |
| 9 | 38 | 0.963 |
| 9 | 44 | 0.794 |
| 9 | 45 | 0.957 |
| 9 | 58 | 0.961 |
| 9 | 66 | 0.984 |
| 9 | 71 | 0.991 |
| 9 | 74 | 0.984 |
| 9 | 78 | 0.928 |
| 9 | 80 | 0.905 |
| 9 | 81 | 0.998 |
| 9 | 83 | 0.958 |
| 9 | 88 | 0.961 |
| 9 | 91 | 0.845 |

| | | |
|---|---|---|
| 9 | 93 | 0.993 |
| 13 | 14 | 0.931 |
| 13 | 17 | 0.919 |
| 13 | 24 | 0.994 |
| 13 | 38 | 0.834 |
| 13 | 44 | 0.938 |
| 13 | 45 | 0.742 |
| 13 | 58 | 0.974 |
| 13 | 66 | 0.989 |
| 13 | 71 | 0.998 |
| 13 | 74 | 0.991 |
| 13 | 78 | 0.888 |
| 13 | 80 | 0.412 |
| 13 | 81 | 0.996 |
| 13 | 83 | 0.728 |
| 13 | 88 | 0.947 |
| 13 | 91 | 0.447 |
| 13 | 93 | 0.968 |
| 14 | 17 | 0.452 |
| 14 | 24 | 0.899 |
| 14 | 38 | 0.903 |
| 14 | 44 | 0.937 |
| 14 | 45 | 0.9 |
| 14 | 58 | 0.873 |
| 14 | 66 | 0.869 |
| 14 | 71 | 0.868 |
| 14 | 74 | 0.894 |
| 14 | 78 | 0.97 |
| 14 | 80 | 0.907 |
| 14 | 81 | 0.852 |
| 14 | 83 | 0.918 |
| 14 | 88 | 0.859 |
| 14 | 91 | 0.952 |
| 14 | 93 | 0.976 |
| 17 | 24 | 0.181 |
| 17 | 38 | 0.84 |
| 17 | 44 | 0.879 |
| 17 | 45 | 0.876 |
| 17 | 58 | 0.147 |
| 17 | 66 | 0.232 |
| 17 | 71 | 0.097 |
| 17 | 74 | 0.163 |
| 17 | 78 | 0.914 |
| 17 | 80 | 0.857 |
| 17 | 81 | 0.26 |
| 17 | 83 | 0.864 |
| 17 | 88 | 0.408 |
| 17 | 91 | 0.915 |
| 17 | 93 | 0.712 |
| 24 | 38 | 0.979 |
| 24 | 44 | 0.95 |
| 24 | 45 | 0.976 |
| 24 | 58 | 0.754 |
| 24 | 66 | 0.719 |
| 24 | 71 | 0.712 |
| 24 | 74 | 0.757 |
| 24 | 78 | 0.99 |
| 24 | 80 | 0.976 |
| 24 | 81 | 0.782 |

| | | |
|---|---|---|
| 24 | 83 | 0.969 |
| 24 | 88 | 0.774 |
| 24 | 91 | 0.993 |
| 24 | 93 | 0.981 |
| 38 | 44 | 0.914 |
| 38 | 45 | 0.512 |
| 38 | 58 | 0.941 |
| 38 | 66 | 0.964 |
| 38 | 71 | 0.979 |
| 38 | 74 | 0.972 |
| 38 | 78 | 0.953 |
| 38 | 80 | 0.733 |
| 38 | 81 | 0.991 |
| 38 | 83 | 0.817 |
| 38 | 88 | 0.945 |
| 38 | 91 | 0.893 |
| 38 | 93 | 0.998 |
| 44 | 45 | 0.919 |
| 44 | 58 | 0.915 |
| 44 | 66 | 0.914 |
| 44 | 71 | 0.913 |
| 44 | 74 | 0.935 |
| 44 | 78 | 0.508 |
| 44 | 80 | 0.929 |
| 44 | 81 | 0.941 |
| 44 | 83 | 0.921 |
| 44 | 88 | 0.933 |
| 44 | 91 | 0.866 |
| 44 | 93 | 0.957 |
| 45 | 58 | 0.957 |
| 45 | 66 | 0.976 |
| 45 | 71 | 0.976 |
| 45 | 74 | 0.981 |
| 45 | 78 | 0.962 |
| 45 | 80 | 0.55 |
| 45 | 81 | 0.995 |
| 45 | 83 | 0.78 |
| 45 | 88 | 0.957 |
| 45 | 91 | 0.775 |
| 45 | 93 | 1 |
| 58 | 66 | 0.644 |
| 58 | 71 | 0.458 |
| 58 | 74 | 0.557 |
| 58 | 78 | 0.963 |
| 58 | 80 | 0.925 |
| 58 | 81 | 0.594 |
| 58 | 83 | 0.949 |
| 58 | 88 | 0.688 |

| | | |
|---|---|---|
| 58 | 91 | 0.971 |
| 58 | 93 | 0.929 |
| 66 | 71 | 0.742 |
| 66 | 74 | 0.782 |
| 66 | 78 | 0.97 |
| 66 | 80 | 0.92 |
| 66 | 81 | 0.417 |
| 66 | 83 | 0.964 |
| 66 | 88 | 0.73 |
| 66 | 91 | 0.982 |
| 66 | 93 | 0.97 |
| 71 | 74 | 0.628 |
| 71 | 78 | 0.973 |
| 71 | 80 | 0.948 |
| 71 | 81 | 0.871 |
| 71 | 83 | 0.988 |
| 71 | 88 | 0.64 |
| 71 | 91 | 0.978 |
| 71 | 93 | 0.985 |
| 74 | 78 | 0.966 |
| 74 | 80 | 0.937 |
| 74 | 81 | 0.729 |
| 74 | 83 | 0.978 |
| 74 | 88 | 0.424 |
| 74 | 91 | 0.978 |
| 74 | 93 | 0.829 |
| 78 | 80 | 0.903 |
| 78 | 81 | 0.983 |
| 78 | 83 | 0.949 |
| 78 | 88 | 0.912 |
| 78 | 91 | 0.704 |
| 78 | 93 | 0.736 |
| 80 | 81 | 0.9 |
| 80 | 83 | 0.427 |
| 80 | 88 | 0.927 |
| 80 | 91 | 0.592 |
| 80 | 93 | 0.913 |
| 81 | 83 | 0.987 |
| 81 | 88 | 0.753 |
| 81 | 91 | 0.993 |
| 81 | 93 | 0.987 |
| 83 | 88 | 0.943 |
| 83 | 91 | 0.777 |
| 83 | 93 | 0.991 |
| 88 | 91 | 0.9 |
| 88 | 93 | 0.273 |
| 91 | 93 | 0.747 |

**Table S14.** Simpson's distances among all clusters resulting from the cluster analysis of genus data (cut-off: 0.75).

| Cluster ID1 | Cluster ID2 | Simpson's distance |
|---|---|---|
| 1 | 2 | 0.442 |
| 1 | 3 | 0.426 |
| 1 | 4 | 0.22 |
| 1 | 5 | 0.236 |
| 1 | 6 | 0.332 |
| 1 | 8 | 0.523 |
| 1 | 9 | 0.256 |
| 1 | 12 | 0.505 |
| 1 | 13 | 0.424 |
| 1 | 17 | 0.25 |
| 1 | 18 | 0.401 |
| 2 | 3 | 0.395 |
| 2 | 4 | 0.516 |
| 2 | 5 | 0.363 |
| 2 | 6 | 0.076 |
| 2 | 8 | 0.327 |
| 2 | 9 | 0.462 |
| 2 | 12 | 0.525 |
| 2 | 13 | 0.371 |
| 2 | 17 | 0.516 |
| 2 | 18 | 0.607 |
| 3 | 4 | 0.315 |
| 3 | 5 | 0.307 |
| 3 | 6 | 0.203 |
| 3 | 8 | 0.399 |
| 3 | 9 | 0.313 |
| 3 | 12 | 0.371 |
| 3 | 13 | 0.248 |
| 3 | 17 | 0.338 |
| 3 | 18 | 0.45 |
| 4 | 5 | 0.093 |
| 4 | 6 | 0.233 |
| 4 | 8 | 0.419 |
| 4 | 9 | 0.523 |
| 4 | 12 | 0.622 |
| 4 | 13 | 0.55 |
| 4 | 17 | 0.536 |
| 4 | 18 | 0.617 |
| 5 | 6 | 0.329 |
| 5 | 8 | 0.469 |
| 5 | 9 | 0.062 |
| 5 | 12 | 0.383 |
| 5 | 13 | 0.248 |
| 5 | 17 | 0.115 |
| 5 | 18 | 0.357 |
| 6 | 8 | 0.136 |
| 6 | 9 | 0.231 |
| 6 | 12 | 0.086 |
| 6 | 13 | 0.023 |
| 6 | 17 | 0.234 |
| 6 | 18 | 0.356 |
| 8 | 9 | 0.385 |
| 8 | 12 | 0.053 |
| 8 | 13 | 0.179 |
| 8 | 17 | 0.338 |
| 8 | 18 | 0.376 |
| 9 | 12 | 0.851 |
| 9 | 13 | 0.862 |
| 9 | 17 | 0.6 |
| 9 | 18 | 0.395 |
| 12 | 13 | 0.725 |
| 12 | 17 | 0.819 |
| 12 | 18 | 0.734 |
| 13 | 17 | 0.758 |
| 13 | 18 | 0.672 |
| 17 | 18 | 0.437 |

**Table S15.** Simpson's distances among all clusters resulting from the cluster analysis of genus data (cut-off: 0.71).

| Cluster ID1 | Cluster ID2 | Simpson's distance |
|---|---|---|
| 1 | 2 | 0.443 |
| 1 | 3 | 0.279 |
| 1 | 4 | 0.197 |
| 1 | 5 | 0.249 |
| 1 | 6 | 0.51 |
| 1 | 10 | 0.523 |
| 1 | 11 | 0.338 |
| 1 | 12 | 0.528 |
| 1 | 13 | 0.31 |
| 1 | 14 | 0.256 |
| 1 | 25 | 0.252 |
| 1 | 26 | 0.38 |
| 1 | 42 | 0.421 |
| 2 | 3 | 0.545 |
| 2 | 4 | 0.504 |
| 2 | 5 | 0.374 |
| 2 | 6 | 0.481 |
| 2 | 10 | 0.326 |
| 2 | 11 | 0.079 |
| 2 | 12 | 0.338 |
| 2 | 13 | 0.46 |
| 2 | 14 | 0.462 |
| 2 | 25 | 0.521 |
| 2 | 26 | 0.561 |
| 2 | 42 | 0.637 |
| 3 | 4 | 0.406 |
| 3 | 5 | 0.206 |
| 3 | 6 | 0.484 |
| 3 | 10 | 0.396 |
| 3 | 11 | 0.238 |
| 3 | 12 | 0.581 |
| 3 | 13 | 0.287 |
| 3 | 14 | 0.379 |
| 3 | 25 | 0.454 |
| 3 | 26 | 0.541 |
| 3 | 42 | 0.579 |
| 4 | 5 | 0.089 |
| 4 | 6 | 0.546 |
| 4 | 10 | 0.417 |
| 4 | 11 | 0.229 |
| 4 | 12 | 0.647 |
| 4 | 13 | 0.5 |
| 4 | 14 | 0.59 |
| 4 | 25 | 0.562 |
| 4 | 26 | 0.651 |
| 4 | 42 | 0.623 |
| 5 | 6 | 0.34 |
| 5 | 10 | 0.476 |
| 5 | 11 | 0.328 |
| 5 | 12 | 0.374 |
| 5 | 13 | 0.168 |
| 5 | 14 | 0.077 |
| 5 | 25 | 0.116 |
| 5 | 26 | 0.3 |
| 5 | 42 | 0.453 |
| 6 | 10 | 0.278 |
| 6 | 11 | 0.1 |
| 6 | 12 | 0.378 |
| 6 | 13 | 0.355 |
| 6 | 14 | 0.431 |
| 6 | 25 | 0.482 |
| 6 | 26 | 0.528 |
| 6 | 42 | 0.62 |
| 10 | 11 | 0.139 |
| 10 | 12 | 0.217 |
| 10 | 13 | 0.365 |
| 10 | 14 | 0.385 |
| 10 | 25 | 0.335 |
| 10 | 26 | 0.338 |
| 10 | 42 | 0.374 |
| 11 | 12 | 0.018 |
| 11 | 13 | 0.18 |
| 11 | 14 | 0.241 |
| 11 | 25 | 0.247 |
| 11 | 26 | 0.309 |
| 11 | 42 | 0.401 |
| 12 | 13 | 0.547 |
| 12 | 14 | 0.626 |
| 12 | 25 | 0.632 |
| 12 | 26 | 0.735 |
| 12 | 42 | 0.789 |
| 13 | 14 | 0.605 |
| 13 | 25 | 0.684 |
| 13 | 26 | 0.758 |
| 13 | 42 | 0.827 |
| 14 | 25 | 0.6 |
| 14 | 26 | 0.405 |
| 14 | 42 | 0.79 |
| 25 | 26 | 0.474 |
| 25 | 42 | 0.681 |
| 26 | 42 | 0.418 |

**Table S16.** Simpson's distances among all clusters resulting from the cluster analysis of genus data (cut-off: 0.67).

| Cluster ID1 | Cluster ID2 | Simpson's distance |
|---|---|---|
| 1 | 2 | 0.454 |
| 1 | 3 | 0.278 |
| 1 | 4 | 0.077 |
| 1 | 5 | 0.293 |
| 1 | 6 | 0.289 |
| 1 | 7 | 0.208 |
| 1 | 8 | 0.51 |
| 1 | 12 | 0.575 |
| 1 | 13 | 0.342 |
| 1 | 14 | 0.531 |
| 1 | 15 | 0.31 |
| 1 | 16 | 0.256 |
| 1 | 22 | 0.516 |
| 1 | 26 | 0.621 |
| 1 | 28 | 0.187 |
| 1 | 31 | 0.252 |
| 1 | 32 | 0.362 |
| 1 | 34 | 0.14 |
| 1 | 46 | 0.564 |
| 1 | 52 | 0.379 |
| 1 | 56 | 0.421 |
| 2 | 3 | 0.565 |
| 2 | 4 | 0.406 |
| 2 | 5 | 0.401 |
| 2 | 6 | 0.492 |
| 2 | 7 | 0.548 |
| 2 | 8 | 0.491 |
| 2 | 12 | 0.35 |
| 2 | 13 | 0.066 |
| 2 | 14 | 0.351 |
| 2 | 15 | 0.472 |
| 2 | 16 | 0.492 |
| 2 | 22 | 0.57 |
| 2 | 26 | 0.494 |
| 2 | 28 | 0.539 |
| 2 | 31 | 0.543 |
| 2 | 32 | 0.583 |
| 2 | 34 | 0.146 |
| 2 | 46 | 0.444 |
| 2 | 52 | 0.58 |
| 2 | 56 | 0.67 |
| 3 | 4 | 0.465 |
| 3 | 5 | 0.221 |
| 3 | 6 | 0.381 |
| 3 | 7 | 0.365 |
| 3 | 8 | 0.484 |
| 3 | 12 | 0.436 |
| 3 | 13 | 0.242 |
| 3 | 14 | 0.583 |
| 3 | 15 | 0.287 |
| 3 | 16 | 0.379 |
| 3 | 22 | 0.588 |
| 3 | 26 | 0.647 |
| 3 | 28 | 0.51 |
| 3 | 31 | 0.454 |
| 3 | 32 | 0.523 |
| 3 | 34 | 0.478 |
| 3 | 46 | 0.54 |
| 3 | 52 | 0.533 |
| 3 | 56 | 0.579 |
| 4 | 5 | 0.297 |
| 4 | 6 | 0.742 |
| 4 | 7 | 0.477 |
| 4 | 8 | 0.677 |
| 4 | 12 | 0.503 |
| 4 | 13 | 0.213 |
| 4 | 14 | 0.723 |
| 4 | 15 | 0.755 |
| 4 | 16 | 0.942 |
| 4 | 22 | 0.51 |
| 4 | 26 | 0.755 |
| 4 | 28 | 0.497 |
| 4 | 31 | 0.71 |
| 4 | 32 | 0.658 |
| 4 | 34 | 0.632 |
| 4 | 46 | 0.948 |
| 4 | 52 | 0.794 |
| 4 | 56 | 0.632 |
| 5 | 6 | 0.029 |
| 5 | 7 | 0.063 |
| 5 | 8 | 0.358 |
| 5 | 12 | 0.469 |
| 5 | 13 | 0.329 |
| 5 | 14 | 0.386 |
| 5 | 15 | 0.175 |
| 5 | 16 | 0.103 |
| 5 | 22 | 0.431 |
| 5 | 26 | 0.417 |
| 5 | 28 | 0.085 |
| 5 | 31 | 0.133 |
| 5 | 32 | 0.307 |
| 5 | 34 | 0.335 |
| 5 | 46 | 0.368 |
| 5 | 52 | 0.379 |
| 5 | 56 | 0.477 |
| 6 | 7 | 0.378 |
| 6 | 8 | 0.422 |
| 6 | 12 | 0.448 |
| 6 | 13 | 0.2 |
| 6 | 14 | 0.562 |
| 6 | 15 | 0.622 |
| 6 | 16 | 0.456 |
| 6 | 22 | 0.546 |
| 6 | 26 | 0.59 |
| 6 | 28 | 0.444 |
| 6 | 31 | 0.702 |
| 6 | 32 | 0.629 |
| 6 | 34 | 0.787 |
| 6 | 46 | 0.888 |
| 6 | 52 | 0.763 |
| 6 | 56 | 0.806 |
| 7 | 8 | 0.508 |

| | | |
|---|---|---|
| 7 | 12 | 0.442 |
| 7 | 13 | 0.225 |
| 7 | 14 | 0.633 |
| 7 | 15 | 0.517 |
| 7 | 16 | 0.6 |
| 7 | 22 | 0.599 |
| 7 | 26 | 0.636 |
| 7 | 28 | 0.54 |
| 7 | 31 | 0.596 |
| 7 | 32 | 0.663 |
| 7 | 34 | 0.688 |
| 7 | 46 | 0.78 |
| 7 | 52 | 0.639 |
| 7 | 56 | 0.652 |
| 8 | 12 | 0.309 |
| 8 | 13 | 0.103 |
| 8 | 14 | 0.376 |
| 8 | 15 | 0.355 |
| 8 | 16 | 0.431 |
| 8 | 22 | 0.528 |
| 8 | 26 | 0.465 |
| 8 | 28 | 0.568 |
| 8 | 31 | 0.482 |
| 8 | 32 | 0.523 |
| 8 | 34 | 0.531 |
| 8 | 46 | 0.36 |
| 8 | 52 | 0.456 |
| 8 | 56 | 0.62 |
| 12 | 13 | 0.083 |
| 12 | 14 | 0.231 |
| 12 | 15 | 0.4 |
| 12 | 16 | 0.472 |
| 12 | 22 | 0.267 |
| 12 | 26 | 0.292 |
| 12 | 28 | 0.522 |
| 12 | 31 | 0.413 |
| 12 | 32 | 0.45 |
| 12 | 34 | 0.42 |
| 12 | 46 | 0.224 |
| 12 | 52 | 0.302 |
| 12 | 56 | 0.474 |
| 13 | 14 | 0.018 |
| 13 | 15 | 0.182 |
| 13 | 16 | 0.251 |
| 13 | 22 | 0.195 |
| 13 | 26 | 0.029 |
| 13 | 28 | 0.297 |
| 13 | 31 | 0.249 |
| 13 | 32 | 0.322 |
| 13 | 34 | 0.125 |
| 13 | 46 | 0 |
| 13 | 52 | 0.266 |
| 13 | 56 | 0.412 |
| 14 | 15 | 0.559 |
| 14 | 16 | 0.646 |
| 14 | 22 | 0.655 |
| 14 | 26 | 0.419 |
| 14 | 28 | 0.704 |
| 14 | 31 | 0.645 |

| | | |
|---|---|---|
| 14 | 32 | 0.759 |
| 14 | 34 | 0.603 |
| 14 | 46 | 0.4 |
| 14 | 52 | 0.686 |
| 14 | 56 | 0.792 |
| 15 | 16 | 0.605 |
| 15 | 22 | 0.585 |
| 15 | 26 | 0.578 |
| 15 | 28 | 0.555 |
| 15 | 31 | 0.684 |
| 15 | 32 | 0.769 |
| 15 | 34 | 0.758 |
| 15 | 46 | 0.808 |
| 15 | 52 | 0.793 |
| 15 | 56 | 0.827 |
| 16 | 22 | 0.528 |
| 16 | 26 | 0.687 |
| 16 | 28 | 0.41 |
| 16 | 31 | 0.6 |
| 16 | 32 | 0.426 |
| 16 | 34 | 0.708 |
| 16 | 46 | 0.928 |
| 16 | 52 | 0.793 |
| 16 | 56 | 0.79 |
| 22 | 26 | 0.627 |
| 22 | 28 | 0.68 |
| 22 | 31 | 0.501 |
| 22 | 32 | 0.457 |
| 22 | 34 | 0.595 |
| 22 | 46 | 0.656 |
| 22 | 52 | 0.284 |
| 22 | 56 | 0.488 |
| 26 | 28 | 0.726 |
| 26 | 31 | 0.679 |
| 26 | 32 | 0.729 |
| 26 | 34 | 0.671 |
| 26 | 46 | 0.244 |
| 26 | 52 | 0.657 |
| 26 | 56 | 0.76 |
| 28 | 31 | 0.352 |
| 28 | 32 | 0.528 |
| 28 | 34 | 0.519 |
| 28 | 46 | 0.72 |
| 28 | 52 | 0.604 |
| 28 | 56 | 0.678 |
| 31 | 32 | 0.499 |
| 31 | 34 | 0.738 |
| 31 | 46 | 0.856 |
| 31 | 52 | 0.58 |
| 31 | 56 | 0.681 |
| 32 | 34 | 0.735 |
| 32 | 46 | 0.908 |
| 32 | 52 | 0.325 |
| 32 | 56 | 0.482 |
| 34 | 46 | 0.88 |
| 34 | 52 | 0.698 |
| 34 | 56 | 0.807 |
| 46 | 52 | 0.911 |
| 46 | 56 | 0.936 |

| | | |
|---|---|---|
| 52 | 56 | 0.272 |

**Table S17.** Simpson's distances among all clusters resulting from the cluster analysis of family data (cut-off: 0.48).

| Cluster ID1 | Cluster ID2 | Simpson's distance |
|---|---|---|
| 1 | 2 | 0.035 |
| 1 | 4 | 0.019 |
| 1 | 5 | 0 |
| 1 | 6 | 0.016 |
| 1 | 10 | 0.045 |
| 1 | 13 | 0.093 |
| 1 | 18 | 0.04 |
| 1 | 19 | 0.018 |
| 1 | 26 | 0.07 |
| 1 | 27 | 0.047 |
| 2 | 4 | 0.011 |
| 2 | 5 | 0.008 |
| 2 | 6 | 0 |
| 2 | 10 | 0 |
| 2 | 13 | 0.016 |
| 2 | 18 | 0 |
| 2 | 19 | 0 |
| 2 | 26 | 0.045 |
| 2 | 27 | 0 |
| 4 | 5 | 0.174 |
| 4 | 6 | 0.148 |
| 4 | 10 | 0.224 |
| 4 | 13 | 0.113 |
| 4 | 18 | 0.23 |
| 4 | 19 | 0.135 |
| 4 | 26 | 0.259 |
| 4 | 27 | 0.304 |
| 5 | 6 | 0.461 |
| 5 | 10 | 0.545 |
| 5 | 13 | 0.098 |
| 5 | 18 | 0.611 |
| 5 | 19 | 0.402 |
| 5 | 26 | 0.167 |
| 5 | 27 | 0.53 |
| 6 | 10 | 0.477 |
| 6 | 13 | 0.039 |
| 6 | 18 | 0.548 |
| 6 | 19 | 0.242 |
| 6 | 26 | 0.172 |
| 6 | 27 | 0.547 |
| 10 | 13 | 0.058 |
| 10 | 18 | 0.413 |
| 10 | 19 | 0.372 |
| 10 | 26 | 0.122 |
| 10 | 27 | 0.541 |
| 13 | 18 | 0.095 |
| 13 | 19 | 0.023 |
| 13 | 26 | 0.066 |
| 13 | 27 | 0.047 |
| 18 | 19 | 0.397 |
| 18 | 26 | 0.206 |
| 18 | 27 | 0.54 |
| 19 | 26 | 0.129 |
| 19 | 27 | 0.446 |
| 26 | 27 | 0.264 |

**Table S18.** Simpson's distances among all clusters resulting from the cluster analysis of family data (cut-off: 0.44).

| Cluster ID1 | Cluster ID2 | Simpson's distance |
|---|---|---|
| 1 | 2 | 0.035 |
| 1 | 4 | 0.016 |
| 1 | 5 | 0.016 |
| 1 | 6 | 0 |
| 1 | 7 | 0.016 |
| 1 | 8 | 0.008 |
| 1 | 13 | 0.045 |
| 1 | 17 | 0.098 |
| 1 | 24 | 0.018 |
| 1 | 34 | 0.07 |
| 1 | 35 | 0.055 |
| 2 | 4 | 0 |
| 2 | 5 | 0.004 |
| 2 | 6 | 0.008 |
| 2 | 7 | 0 |
| 2 | 8 | 0.017 |
| 2 | 13 | 0 |
| 2 | 17 | 0.016 |
| 2 | 24 | 0 |
| 2 | 34 | 0.045 |
| 2 | 35 | 0 |
| 4 | 5 | 0.244 |
| 4 | 6 | 0.167 |
| 4 | 7 | 0.102 |
| 4 | 8 | 0.252 |
| 4 | 13 | 0.269 |
| 4 | 17 | 0.082 |
| 4 | 24 | 0.16 |
| 4 | 34 | 0.271 |
| 4 | 35 | 0.172 |
| 5 | 6 | 0.235 |
| 5 | 7 | 0.156 |
| 5 | 8 | 0.168 |
| 5 | 13 | 0.237 |
| 5 | 17 | 0.102 |
| 5 | 24 | 0.16 |
| 5 | 34 | 0.252 |
| 5 | 35 | 0.352 |
| 6 | 7 | 0.461 |
| 6 | 8 | 0.42 |
| 6 | 13 | 0.545 |
| 6 | 17 | 0.098 |
| 6 | 24 | 0.402 |
| 6 | 34 | 0.167 |
| 6 | 35 | 0.538 |
| 7 | 8 | 0.588 |
| 7 | 13 | 0.477 |
| 7 | 17 | 0.039 |
| 7 | 24 | 0.242 |
| 7 | 34 | 0.18 |
| 7 | 35 | 0.555 |
| 8 | 13 | 0.504 |
| 8 | 17 | 0.092 |
| 8 | 24 | 0.395 |
| 8 | 34 | 0.151 |
| 8 | 35 | 0.681 |
| 13 | 17 | 0.058 |
| 13 | 24 | 0.372 |
| 13 | 34 | 0.122 |
| 13 | 35 | 0.545 |
| 17 | 24 | 0.018 |
| 17 | 34 | 0.066 |
| 17 | 35 | 0.048 |
| 24 | 34 | 0.13 |
| 24 | 35 | 0.441 |
| 34 | 35 | 0.276 |

## Supporting References


1. Costello, M. J. *et al.* Marine biogeographic realms and species endemicity. *Nat. Commun.* **8**, 1057 (2017).
2. Kocsis, Á. T., Reddin, C. J. & Kiessling, W. The stability of coastal benthic biogeography over the last 10 million years. *Glob. Ecol. Biogeogr.* **27**, 1106–1120 (2018).
3. GBIF.org. GBIF Occurrence Download. https://doi.org/10.15468/dl.gtxbs9 (2024).
4. OBIS. Global distribution records of Mollusca. Available: Ocean Biodiversity Information System. Intergovernmental Oceanographic Commission of UNESCO. https://obis.org (2024).
5. Cao, M. *et al.* A new global gridded sea surface temperature data product based on multisource data. *Earth Syst. Sci. Data* **13**, 2111–2134 (2021).
6. Spalding, M. D. *et al.* Marine Ecoregions of the World: A Bioregionalization of Coastal and Shelf Areas. *BioScience* **57**, 573–583 (2007).
