# Supplementary material for: Biogeographic patterns of modern benthic shallow-water molluscs and the roles of temperature and palaeogeographic legacy
Source: Sci Rep. 2025 Jul 1;15:20304. doi: 10.1038/s41598-025-06473-0 (PMC12217645; doi:10.1038/s41598-025-06473-0)

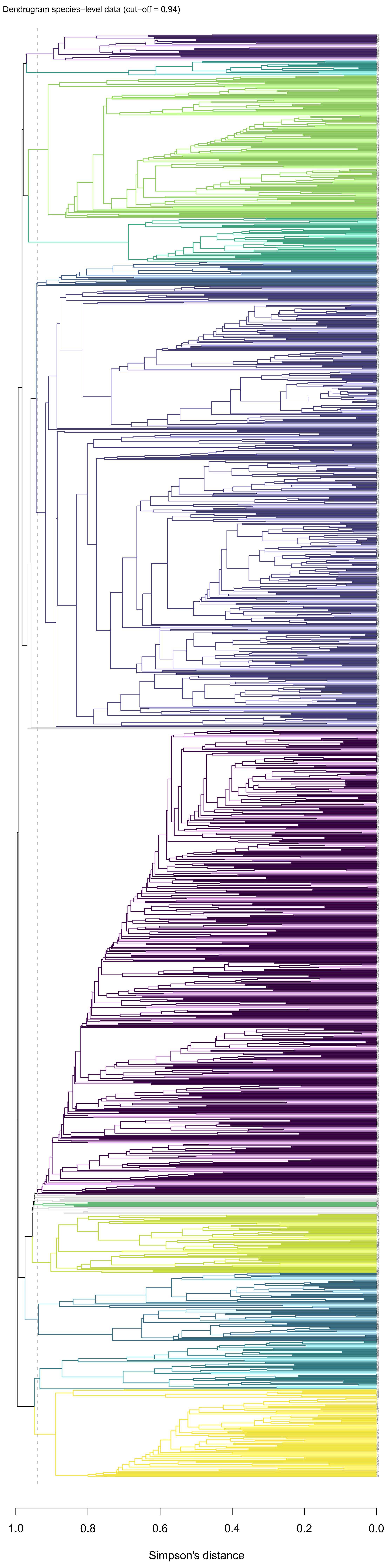

Dendrogram species−level data (cut−off = 0.94)
1.0
0.8
0.6
0.4
0.2
0.0
Simpson's distance

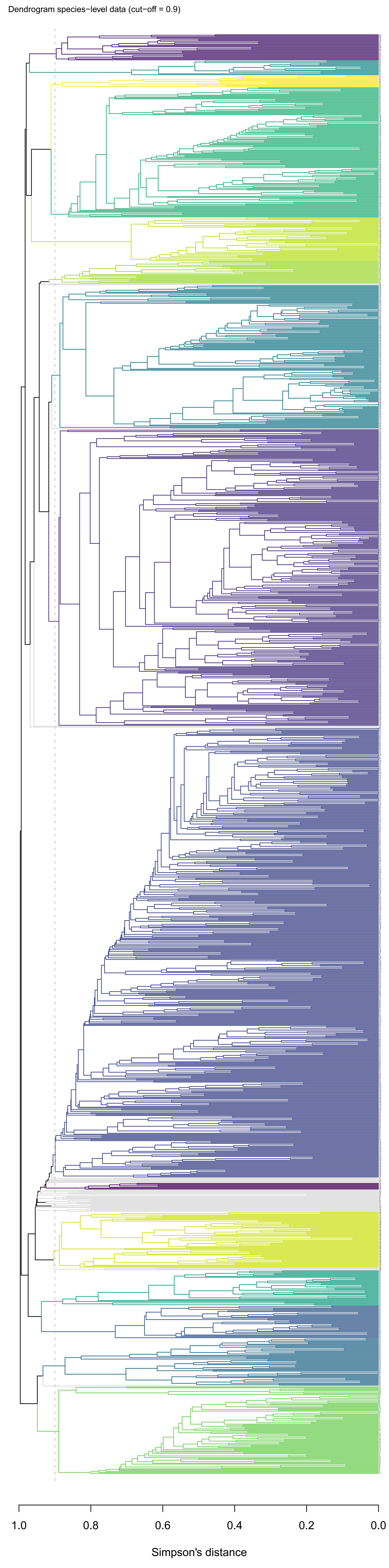

Dendrogram species−level data (cut−off = 0.9)
1.0
0.8
0.6
0.4
0.2
0.0
Simpson's distance

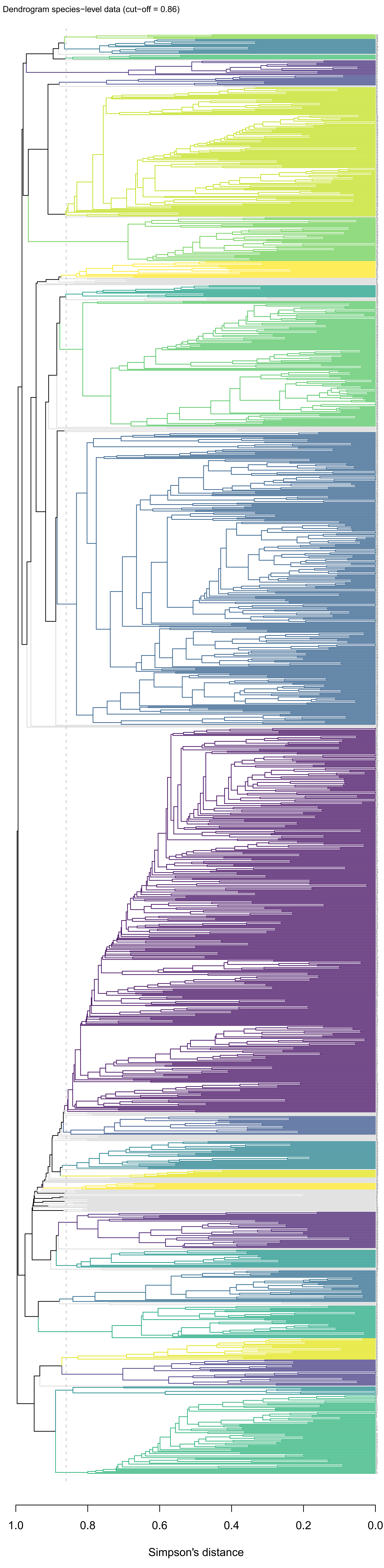

Dendrogram species−level data (cut−off = 0.86)
1.0
0.8
0.6
0.4
0.2
0.0
Simpson's distance

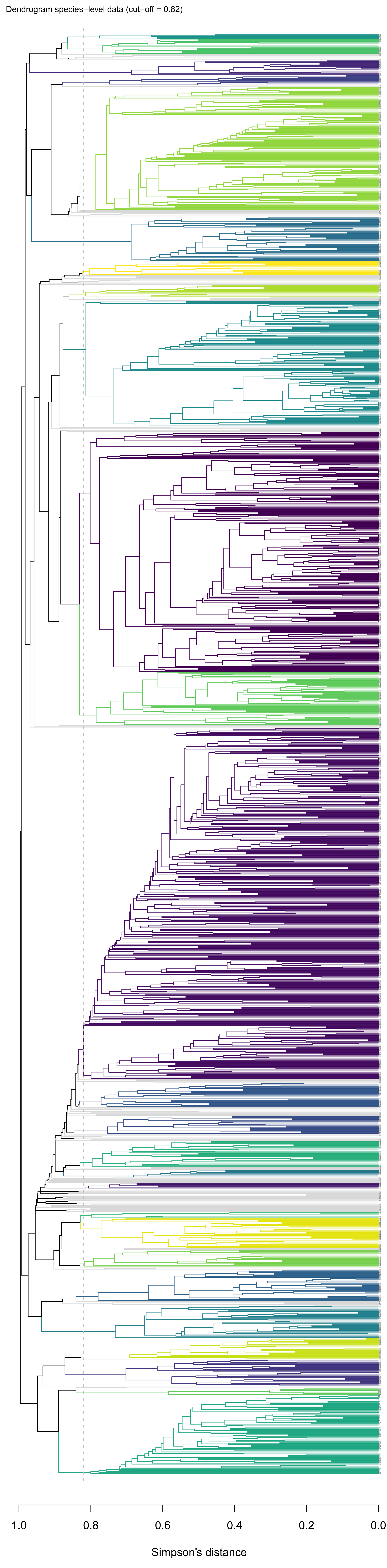
Dendrogram species−level data (cut−off = 0.82)
1.0
0.8
0.6
0.4
0.2
0.0
Simpson's distance

Dendrogram genus−level data (cut−off = 0.75)

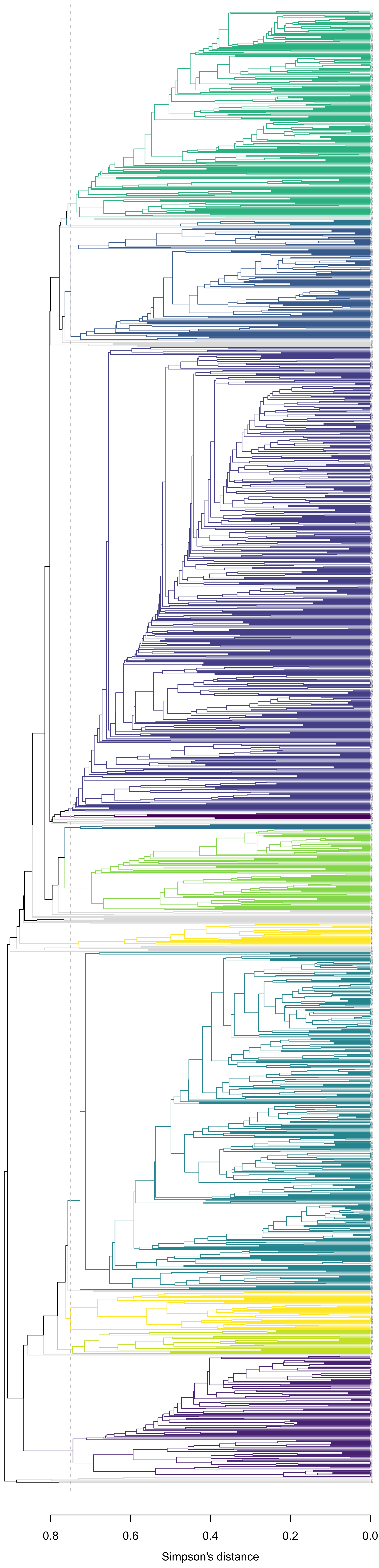

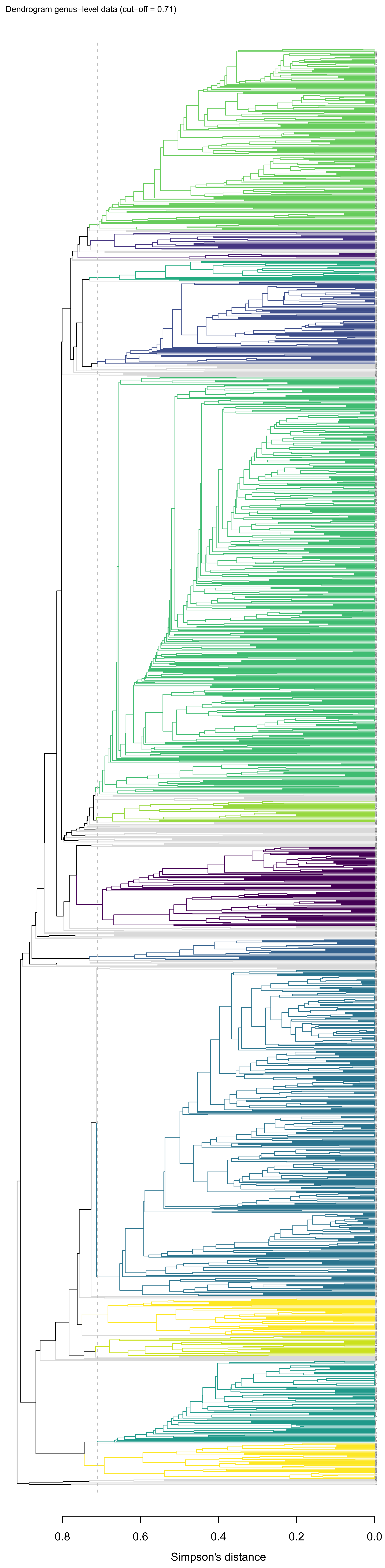
Dendrogram genus−level data (cut−off = 0.71)
0.8
0.6
0.4
0.2
0.0
Simpson's distance

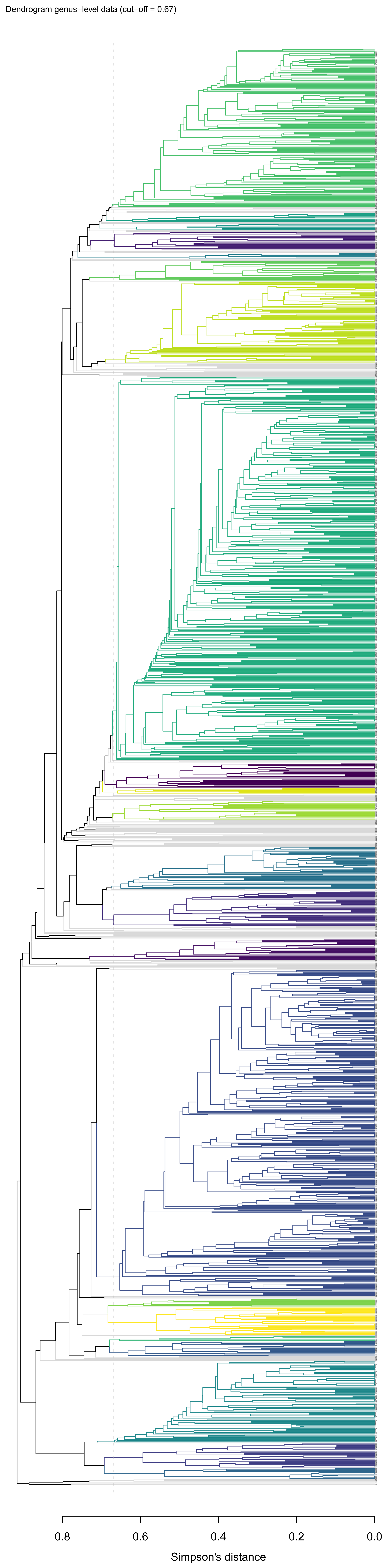
Dendrogram genus−level data (cut−off = 0.67)
0.8
0.6
0.4
0.2
0.0
Simpson's distance

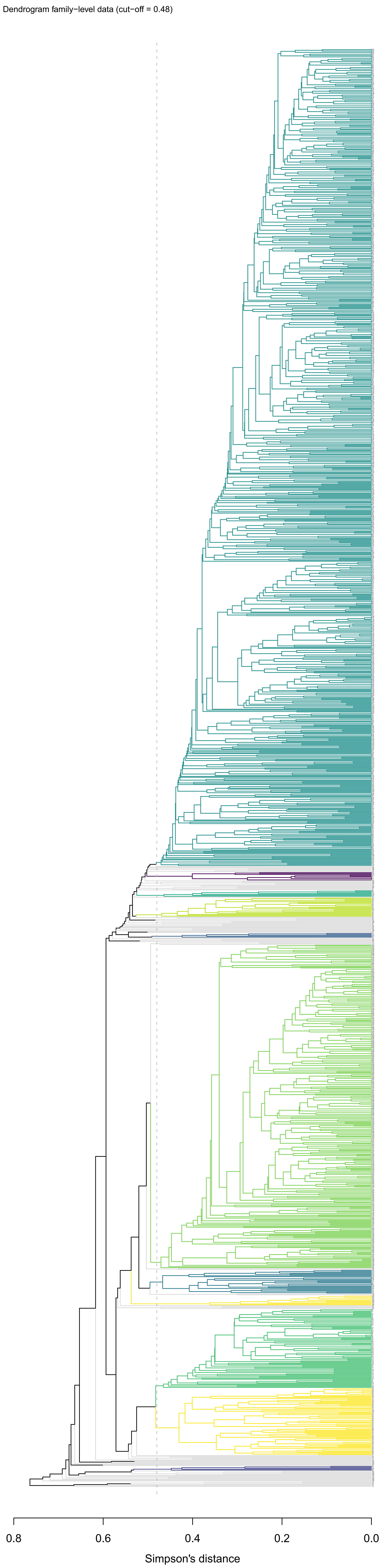
Dendrogram family−level data (cut−off = 0.48)
0.8
0.6
0.4
0.2
0.0
Simpson's distance

Dendrogram family−level data (cut−off = 0.44)

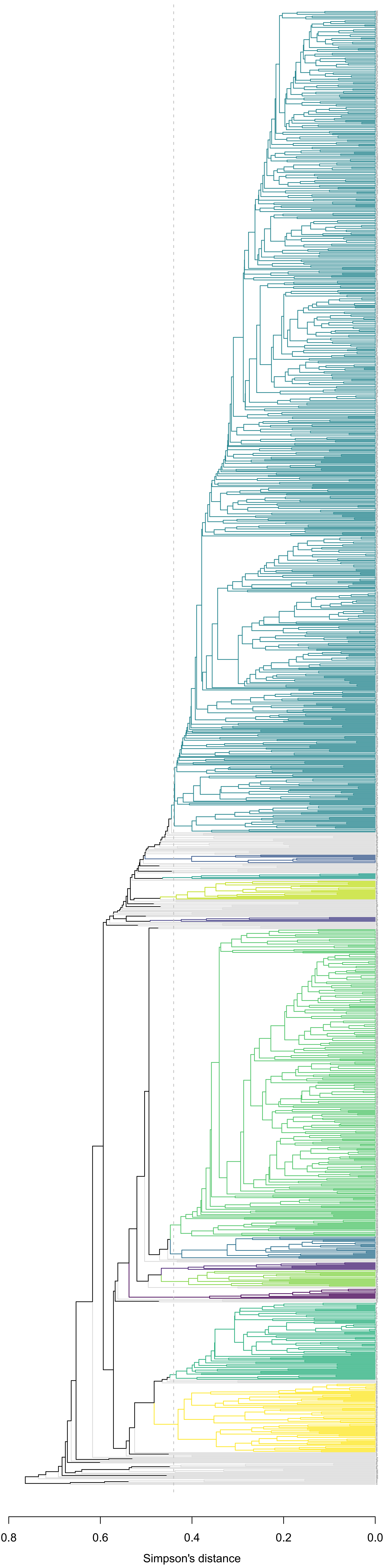

Supplement: Supplementary file 2 — Supplementary Information 2. [file 41598_2025_6473_MOESM2_ESM.pdf]
